# Pleiotropic Effects of PhaR Regulator in *Bradyrhizobium diazoefficiens* Microaerobic Metabolism

**DOI:** 10.3390/ijms25042157

**Published:** 2024-02-10

**Authors:** Juan I. Quelas, Juan J. Cabrera, Rocío Díaz-Peña, Lucía Sánchez-Schneider, Andrea Jiménez-Leiva, Germán Tortosa, María J. Delgado, M. Julia Pettinari, Aníbal R. Lodeiro, Coral del Val, Socorro Mesa

**Affiliations:** 1 Instituto de Biotecnología y Biología Molecular, Facultad de Ciencias Exactas, Universidad Nacional de La Plata y CCT-La Plata, CONICET, La Plata 1900, Argentina; juan.i.quelas@gmail.com (J.I.Q.); lodeiro@biol.unlp.edu.ar (A.R.L.); 2 YPF Tecnología S.A. (Y-TEC), Avenida. del Petróleo Argentino s/n (1923), Berisso 1923, Argentina; 3 Department of Soil and Plant Microbiology, Estación Experimental del Zaidín, CSIC, 18008 Granada, Spain; juan.cabrera@eez.csic.es (J.J.C.); luciasanchez6@hotmail.com (L.S.-S.); andrea.jimenez@eez.csic.es (A.J.-L.); german.tortosa@eez.csic.es (G.T.); mariajesus.delgado@eez.csic.es (M.J.D.); 4 IQUIBICEN-CONICET, Facultad de Ciencias Exactas y Naturales, Universidad de Buenos Aires, Intendente Güiraldes, C1428EHA, CABA, Buenos Aires 2160, Argentina; rociodiazpena@gmail.com (R.D.-P.); jul@qb.fcen.uba.ar (M.J.P.); 5 Departamento de Química Biológica, Facultad de Ciencias Exactas y Naturales, Universidad de Buenos Aires, Intendente Güiraldes, C1428EHA, CABA, Buenos Aires 2160, Argentina; 6 Department of Computer Science and Artificial Intelligence, Andalusian Research Institute in Data Science and Computational Intelligence (DaSCI), University of Granada, 18016 Granada, Spain; delval@ugr.es; 7 Cátedra de Genética, Facultad de Ciencias Agrarias y Forestales, Universidad Nacional de La Plata, La Plata 1900, Argentina

**Keywords:** microoxia, polyhydroxyalkanoate, protein–DNA interaction, proteomics, rhizobia, transcriptomics

## Abstract

*Bradyrhizobium diazoefficiens* can live inside soybean root nodules and in free-living conditions. In both states, when oxygen levels decrease, cells adjust their protein pools by gene transcription modulation. PhaR is a transcription factor involved in polyhydroxyalkanoate (PHA) metabolism but also plays a role in the microaerobic network of this bacterium. To deeply uncover the function of PhaR, we applied a multipronged approach, including the expression profile of a *phaR* mutant at the transcriptional and protein levels under microaerobic conditions, and the identification of direct targets and of proteins associated with PHA granules. Our results confirmed a pleiotropic function of PhaR, affecting several phenotypes, in addition to PHA cycle control. These include growth deficiency, regulation of carbon and nitrogen allocation, and bacterial motility. Interestingly, PhaR may also modulate the microoxic-responsive regulatory network by activating the expression of *fixK_2_* and repressing *nifA*, both encoding two transcription factors relevant for microaerobic regulation. At the molecular level, two PhaR-binding motifs were predicted and direct control mediated by PhaR determined by protein-interaction assays revealed seven new direct targets for PhaR. Finally, among the proteins associated with PHA granules, we found PhaR, phasins, and other proteins, confirming a dual function of PhaR in microoxia.

## 1. Introduction

Soil bacteria of the genus *Bradyrhizobium* spp., which can live inside soybean root nodules and under free-living conditions, accumulate polyhydroxyalkanoates (PHAs) in both states [1]. In particular, *B. diazoefficiens* USDA 110 synthesizes the most common type of PHA, called poly-3-hydroxybutyrate (PHB) [2]. This polymer not only acts as an intracellular carbon reserve in both free-living and symbiotic states, but also as a redox regulator and influences competitiveness for nodule colonization on soybean nodules [1,2,3]. The genome of *B. diazoefficiens* USDA 110 contains all the genes needed for PHB metabolism, including regulators [4]. Moreover, most of them are present in more than one copy, in an apparent genetic redundancy.

PHB synthesis begins with a reaction catalyzed by β-ketothiolase (PhaA) that condenses two acetyl coenzyme A (acetyl-CoA) moieties to acetoacetyl-CoA, followed by reduction of acetoacetyl-CoA to hydroxybutyryl-CoA (with NADPH consumption) catalyzed by acetoacetyl-CoA reductase (PhaB), and finally polymerization of hydroxybutyryl-CoA into PHB catalyzed by PHB synthase (PhaC). These three enzymes have putative paralogs in the *B. diazoefficiens* genome [2,5]. Two open reading frames (ORFs) encode PhaA (designated PhaA1 and PhaA2), two for PhaB (PhaB1 and PhaB2), and five for PhaC (PhaC1-5) [1]. In a previous work with single mutants of the five *phaC* paralogs, we showed that PhaC1 is the only enzyme required for PHB polymerization. However, since *phaC1* and *phaC2* were transcriptionally active, we proposed a role for PhaC2 that regulates PHB synthase activity through the relative abundance of PhaC2 in PhaC1/PhaC2 heterodimers [1]. Depolymerization is mediated by PHB depolymerase (PhaZ), which is encoded by four putative ORFs (PhaZ1-4).

PHB metabolism is regulated by two key players: PhaR, which encodes a PHA repressor, and PhaP (phasins). PhaR is a conserved and well-studied transcription factor in different bacterial classes, including Archaea [6], modulating PHB synthesis by binding to the promoters of phasin-encoding genes and also to the PHB granules surface [7,8]. Like in all α-proteobacteria, *B. diazoefficiens* USDA 110 PhaR possesses two conserved domains, namely, PHB_acc_n (PF07879) and PHB_acc (PF05233) [6]. These two domains are important for binding to DNA and to PHB granules, respectively [9]. Thus, PhaR has a dual function depending on PHB accumulation and growth conditions. However, the molecular mechanism of PhaR has not been investigated in detail.

Phasins are small amphiphilic proteins that also regulate PHB synthesis, and affect the volume/surface ratio of the granules [10,11]. These heterogeneous proteins bind to PHB and prevent granule coalescence [12]. Furthermore, they also have a diverse function comprising granule morphology regulation, PHB degradation, activation of PHB synthases expression and activity, and chaperone activity [13,14]. Phasins have also been reported to bind to the N-terminal domain of PhaC [15] and to the *phaC* promoter in *Aeromonas hydrophyla* [16]. Bacterial genomes typically contain multiple phasin gene paralogs, which may indicate their functional diversity and versatility [10,12].

The *B. diazoefficiens* USDA 110 genome contains at least seven ORFs encoding phasin paralogs (PhaP1-7) ([5], this work). In this sense, the PhaR regulator exerts a negative control on PHB-related genes, and phasins PhaP1 and PhaP4 are involved in regulating PHB granule size and accumulation in aerobic free-living cultures [2]. Since *B. diazoefficiens* can accumulate high levels of PHB in both free-living and symbiotic states [1,2], it is important to understand the metabolic regulation of PHB synthesis and degradation pathways. In this species, the excess of carbon source increases exopolysaccharide (EPS) or polyhydroxybutyrate (PHB) production, depending on N availability.

When bacterial cells grow at a high C/N ratio, carbon is diverted to PHB accumulation instead of EPS [2,17]. Furthermore, PHB accumulation has also been observed in *B. diazoefficiens* under oxygen-limiting conditions (e.g., microoxia, oxic stationary phase, and symbiotic bacteroids inside soybean nodules), so-called permissive conditions. In this regard, *phaR* mutation affects PHB synthesis through gene repression but also impairs growth kinetics when cells are grown in microoxia with mannitol as the sole carbon source. PhaR also represses (directly or indirectly) the expression of the *fixK*_2_ gene encoding a cyclic AMP receptor protein/fumarate and nitrate reduction regulator (CRP/FNR)-type transcription factor, which forms part of the microoxic-responsive regulatory network comprising two interlinked regulatory cascades: FixLJ-FixK_2_-NnrR and RegSR-NifA [2,17]. These findings led us to propose PhaR as a transcription factor with a fine-tuning function in PHB metabolism, depending on the stage of PHB granule growth, and also with a global regulatory role under microaerobic conditions [2].

To deepen our understanding about the function of PhaR in *B. diazoefficiens* microaerobic lifestyle, in this work, we performed a global approach including transcriptomic and proteomic studies in cells grown under permissive conditions for PHB synthesis (i.e., microoxia with mannitol as carbon source), bioinformatics to predict a PhaR-binding site, protein–DNA interaction assays to identify direct targets for PhaR, and the identification of PHB-granule-associated proteins. Taken together, these results expand the pleiotropic role of PhaR in global carbon and nitrogen allocation, bacterial physiology and motility, and microaerobic regulation, in addition to its regulatory role in PHB balance.

## 2. Results

### 2.1. Mutation in the phaR Gene Affects Growth Rate, PHB Accumulation, RNA Levels, and Protein Abundance under Microaerobic Conditions

**Figure 1:**
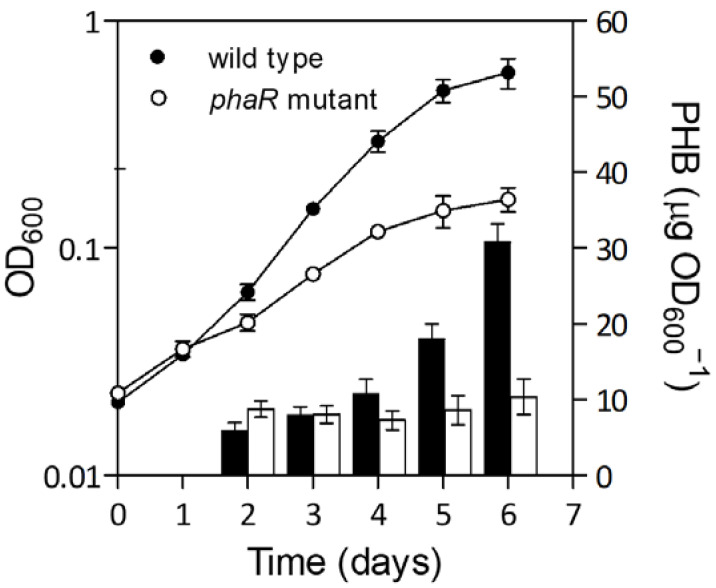
Growth kinetics (dots) and PHB levels (bars) of wild type and the *phaR* mutant cultivated microoxically. Cells were grown in Götz minimal medium supplemented with mannitol under microaerobic conditions (0.5% O_2_). Black dots and bars, wild type; white dots and bars, *phaR* mutant.

As mentioned above, the influence of PhaR is mainly exerted under permissive conditions for PHB production, such as microoxia [2]. In this environment, PHB accumulation and total cell biomass (OD_600_) were affected in a *phaR* mutant, as it exhibited a lower growth yield from the beginning of the culture than the wild type, and lower PHB production monitored within seven days of growth in Götz minimal medium with mannitol under microaerobic conditions (Figure 1).

**Figure 2:**
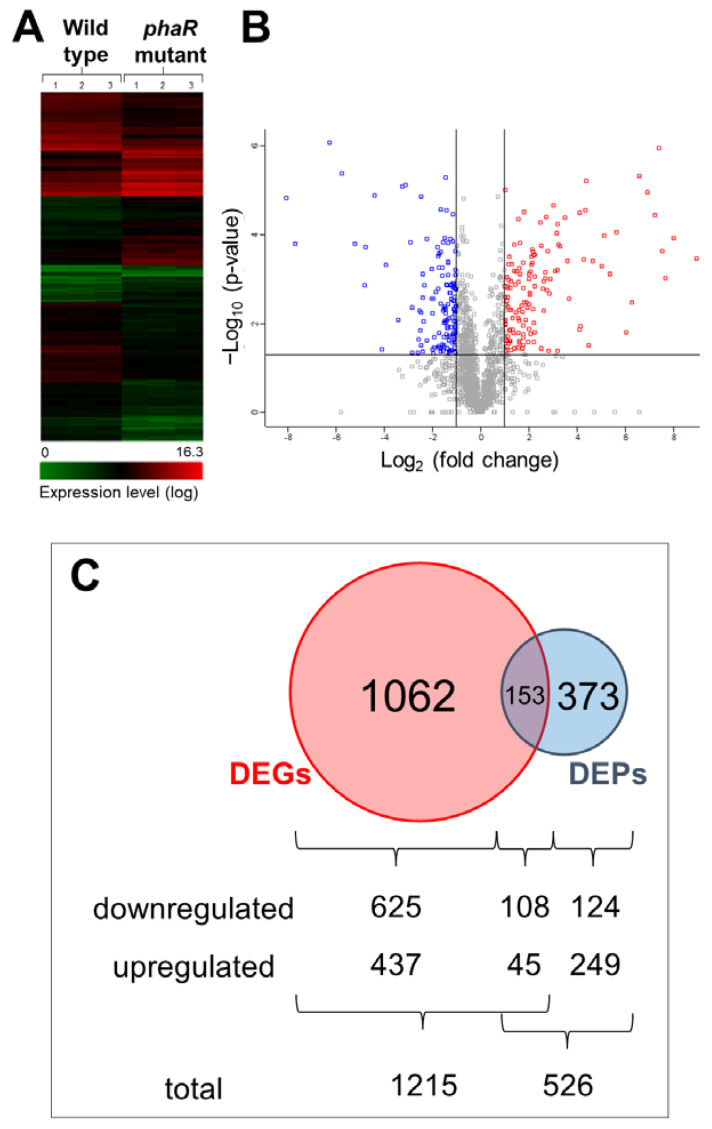
PhaR-mediated regulation at transcriptional and protein levels. (**A**) Hierarchical clustering of gene expression changes detected in transcriptomic experiments. The color code for the expression level is shown at the bottom; red represents a high expression level and green a low expression level (log scale). (**B**) Volcano plot of differentially expressed proteins identified by proteomics; blue represents low abundance; red represents high abundance; grey represents proteins that did not pass either the fold change or the *p*-value threshold or both. (**C**) Venn diagram showing the overlap of differentially expressed genes (DEGs, red circle) compared to differentially expressed proteins (DEPs, blue circle) in the *phaR* mutant in comparison with the wild type. Both strains were grown in Götz minimal medium supplemented with mannitol under microaerobic conditions (0.5% O_2_). The number of downregulated and upregulated DEGs and DEPs in each sector of the Venn diagram is given at the bottom of the panel.

To obtain a more comprehensive picture of the PhaR function, we performed a differential expression profiling at the transcriptional and protein levels of a *phaR* mutant compared to the wild-type strain, both grown in Götz minimal medium with mannitol as the sole carbon source under microaerobic conditions. Figure 2 shows the hierarchical clustering of genes identified by transcriptomics (Figure 2A) and the volcano plot of differentially expressed proteins (DEPs, 526 proteins; Figure 2B). We detected 1215 differentially expressed genes (DEGs), 733 with downregulated expression and 482 with upregulated expression, in the *phaR* mutant (Table S1). In the proteomic approach, among the group of 526 DEPs, 232 were downregulated and 294 were upregulated in the *phaR* mutant compared to the wild type (Figure 2C; Table S2). The overlap between DEGs and DEPs resulted in a set of 153 genes/proteins (45 downregulated and 108 upregulated) (Figure 2C), of which 95% shared the same regulation profile at the transcriptional and protein levels (up- or downregulation), with only 9 outliers (5%) showing an inverse regulation.

### 2.2. PhaR Negatively Controls PHB Metabolism

To evaluate the role of PhaR in PHB cycling in more detail, we searched for all PHB-related paralogs with a differential expression in the *phaR* mutant versus the wild type with a relative change of expression (fold change, FC) ≥ 2 for repressed genes, and ≤−2 for activated genes) (Table S1). Microarray-based transcriptomics confirmed previous results reported by Quelas and coworkers [2], and expanded the function of PhaR in the regulation of PHB-related genes.

**Figure 3:**
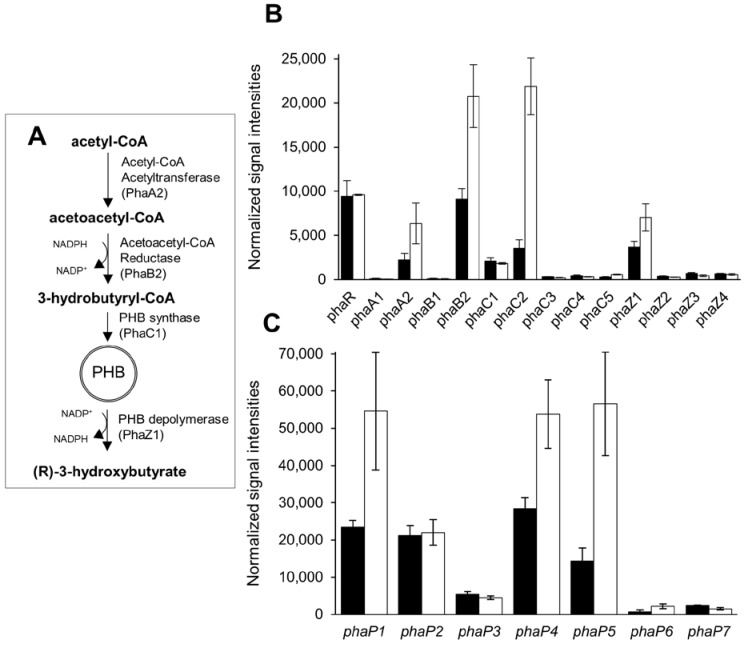
PhaR control on PHB-related genes in *B. diazoefficiens*. (**A**) Schematic representation of PHB metabolism in *B. diazoefficiens* under microaerobic conditions. (**B**,**C**) Normalized signal intensities of microarray data showing genes related to PHB metabolism (**B**) and phasins (**C**) in wild-type (black bars) and *phaR* mutant (white bars) cells, both grown in Götz minimal medium supplemented with mannitol under microaerobic conditions (0.5% O_2_). Values are means ± standard errors of three independent biological replicates per strain. Note that in this study, bll4997 was renamed as *phaZ4* encoding a PHB depolymerase based on sequence similarity with other bacteria.

Among the genes encoding PHB synthesis and degradation enzymes, we found five genes repressed by PhaR with an FC ≥ 2, namely, *phaA2* (bll0226), *phaB2* (bll0225), *phaC2* (bll6073), *phaC5* (bll4548), and *phaZ1* (blr0908). In this regard, PhaR controls one paralog of the first step of PHB synthesis (*phaA2*), one of the second step (*phaB2*), two of the third step (*phaC2* and *phaC5*), and one of the degradation step (*phaZ1*). Figure 3A shows a schematic representation of the PHB pathway in *B. diazoefficiens*, with the specific paralog associated with each step under microaerobic conditions ([2], this study).

Interestingly, *phaC2* has the highest expression and it was strongly repressed by PhaR (FC = 6.8 in the *phaR* mutant vs. wild type), while *phaC5* is the PhaR-regulated gene with the lowest expression levels (Figure 3B). The *phaC1* gene encoding the main PHB polymerase involved in the third step of PHB biosynthesis was not regulated by PhaR, and was also moderately expressed in both strains under the growth conditions tested (Figure 3B), suggesting that PHB metabolism remains active in the *phaR* mutant [1]. The other paralogs of the PHB cycle that are not subject to PhaR-mediated control, namely, *phaA1* (blr3724), *phaB1* (blr3725), *phaZ2* (blr6703), *phaZ3* (blr0899), *phaZ4* (bll4997), *phaC3* (blr3732), and *phaC4* (blr2885), were poorly expressed or even absent in both the wild-type and the mutant strains (Figure 3B). These genes may represent evolutionary transformed pseudogenes and/or genes that require different culture conditions to be expressed. Regarding proteomics, in line with the transcriptomic data, only peptides of PhaA2, PhaB2, and PhaZ1 were detected. While PhaA2 and PhaB2 showed no statistical differences between *phaR* mutant and wild type, PhaZ1 peptides were found only in the *phaR* mutant, thus reinforcing the idea that PHB is degraded at a higher rate in this strain. On the other hand, no peptides related to any of the predicted PHB synthases were detected.

Seven predicted phasin genes are present in *B. diazoefficiens* ([5,18], this work): *phaP1* (bll5155), *phaP2* (bll5555), *phaP3* (bll6129), *phaP4* (bll7395), *phaP5* (blr2887), *phaP6* (blr3464), and *phaP7* (bll5940). Among them, four appeared to be repressed by PhaR, i.e., *phaP1*, *phaP4*, *phaP5*, and *phaP6* (Table S1), while the other three (*phaP2*, *phaP3,* and *phaP7*) appeared to be moderately transcribed under our microaerobic conditions (Figure 3C). Moreover, *phaP3*, *phaP6*, and *phaP7* are phasin genes with low signal intensities in the wild-type strain (*phaP3* > *phaP7* > *phaP6*), compared to the other four phasin paralogs with higher expression (Figure 3C). In our proteomic analysis, three of these seven predicted phasins showed a differential expression in the *phaR* mutant: PhaP1 (Bll5155) and PhaP5 (Blr2887), with positive log_2_ FCs of 0.9 and 0.7, respectively (although they did not pass the *p*-value threshold of 0.05), and PhaP2 (Bll5555) with a negative log_2_ FC = −2.7 (Table S2). This result suggested that *phaP2*/PhaP2 might be subject to a different type of control than that mediated by PhaR.

### 2.3. PhaR Regulates Both the FixK_2_ and NifA Regulons

**Figure 4:**
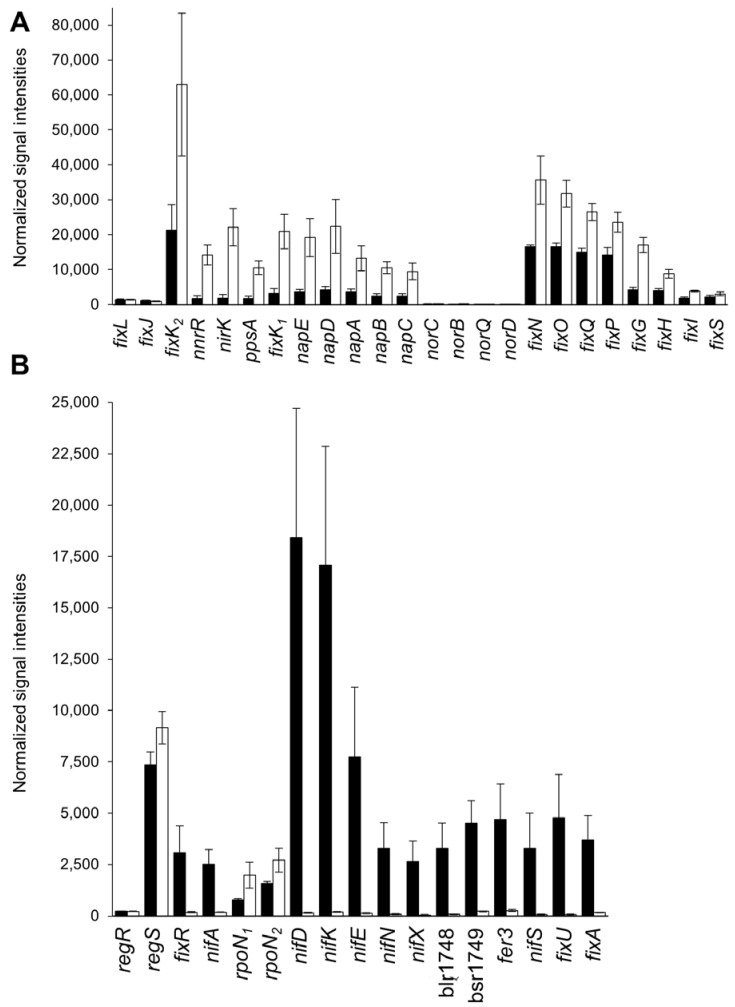
Effect of *phaR* mutation on the low-oxygen responsive network and associated genes in *B. diazoefficiens*. Normalized signal intensities of microarray data showing genes related to the RegSR-NifA cascade (**A**) or the FixLJ-FixK_2_ cascade (**B**) of wild-type (black bars) and *phaR* mutant (white bars) cells, both grown in Götz minimal medium supplemented with mannitol under microaerobic conditions (0.5% O_2_). Values are means ± standard errors of three independent biological replicates per strain.

As previously reported by qRT-PCR experiments [2], our transcriptomic analysis showed that *fixK_2_* gene expression was slightly induced (FC = 3.0) in the *phaR* mutant compared to the wild type (Table S1). This type of PhaR regulation on *fixK_2_* (i.e., repression) was also extended to recognized FixK_2_ targets [17,19], while the superimposed FixLJ system encoding genes remained unaffected (Figure 4A). In particular, the expression of FixK_2_ targets such as *fixN* (blr2763), *fixG* (blr2767), *nnrR* (blr7084), *ppsA* (blr4655), *hemN_2_* (bll7086), *phaC2* (bll6073), *napE* (bsr7036), and *nirK* (blr7089), among others, was induced with relative changes in expression ranging from two-fold to thirteen-fold in the *phaR* mutant compared to the wild type (Table S1), which correlated with the normalized signal intensity levels (Figure 4A). None of the corresponding gene products were detected in the proteomic analyses, with the exception of NnrR, which had slightly higher abundance (log_2_ FC = 0.8) in the *phaR* mutant than in the wild type (with a *p*-value slightly higher than 0.05). Specifically, we found that 80% (41 out of 51) of the putative FixK_2_ direct targets [17] and that 94% (17 out of 18) of the direct targets that were confirmed in a FixK_2_-mediated in vitro transcription (IVT) activation assay [19] showed higher expression in the *phaR* mutant compared to the wild type.

In contrast to *fixK_2_*, the *fixR*-*nifA* operon, encoding the nitrogen fixation regulator NifA, which is part of the RegSR-NifA regulatory cascade [20], showed a downregulated expression in the *phaR* mutant (FC = −11.9 and −13.5 for *fixR* and *nifA*, respectively) (Table S1).

Such a similar expression profile was found for 52 out of 65 (80%) targets of NifA [20], whose expression was reduced in the *phaR* mutant compared to the wild type, but not for the genes encoding the superimposed RegSR two-component regulatory system [21] (Figure 4B shows the expression profile of a selection of NifA targets). Consistent with the observed transcriptional profile, the analysis of the proteomic experiments revealed peptides for FixR (Blr2036), NifH (Blr1769), and NifK (Blr1744) exclusively in wild-type cells (Table S2). Note that the expression of the two RNA polymerase σ^54^ factors genes *rpoN_1_* (a target of FixK_2_) and *rpoN_2_* are increased in the *phaR* mutant (Figure 4B), although they collaborate with NifA for the activation of target genes [20].

### 2.4. PhaR Controls Carbon and Nitrogen Allocation of Key Metabolic Central Pathways

In addition to the described function of PhaR in PHB cycle balance and its role as a modulator of the microaerobic metabolism in *B. diazoefficiens*, the entire operon and their corresponding protein products involved in mannitol acquisition and oxidation were found to be downregulated in the *phaR* mutant (Table S3). Specifically, the four genes required for mannitol transport across the membrane and their conversion to fructose-6-P, namely, blr3224, coding for putative mannitol transport system; blr3225, coding for mannitol dehydrogenase; blr3226, coding for ribitol kinase and blr3227; coding for D-fructokinase were downregulated at transcriptional level (Tables S1 and S3). Of these four proteins, Blr3225 and Blr3226 were also less abundant in the *phaR* mutant at protein level (Table S3). In addition, we identified three putative periplasmic mannitol-binding proteins. Two of them (Blr3745 and Blr7816 paralogs) were also downregulated in the *phaR* mutant cells, and one (Blr3743) was more abundant (log_2_ FC = 0.8) (Table S3). This last periplasmic mannitol-binding protein paralog might be responsible for mannitol uptake from the culture medium in the *phaR* mutant.

In a previous study by Cogo and coworkers [22], it was reported that mannitol catabolism in *B. diazoefficiens* occurs via the pentose phosphate (PP) and Calvin–Benson–Bassham (CBB) cycles rather than via the Embden–Meyerhof–Parnas (EMP) pathway. Regarding the CBB cycle, we found that the entire *cbb* operon (blr2581 to blr2588) was expressed under the growth conditions of our assays (microoxia in the presence of mannitol), but was downregulated in the *phaR* mutant (Table S3). In this line, the gene products phoshoribulokinase (CbbP; EC 2.7.1.19) and ribulose bisphosphate carboxylase oxygenase (CbbL; EC 4.1.1.39), both specific for the CBB pathway, were found exclusively in the wild type (Table S3). Therefore, we concluded that the CBB pathway is not active in the *phaR* mutant. Interestingly, we found a strong upregulation of the blr4659 gene that encodes phosphofructokinase (PfkB, EC 2.7.1.11), a key enzyme of the EMP pathway, in *phaR* mutant cells (Table S3). Furthermore, two glucokinases (EC 2.7.1.2) encoded by blr4658 and blr5550 genes also showed an increased abundance in the *phaR* mutant (Table S3). These two latter pieces of evidence indicate that the preparatory phase of glycolysis is carried out through the EMP pathway in the *phaR* mutant, although it was considered inactive in *B. diazoefficiens* [23].

Another group of upregulated genes in the *phaR* mutant are related to branched-chain amino acids degradation (Table S3). The *bkdA1* and *bkdA2* genes encoding the α and β subunits of the 2-oxoisovalerate dehydrogenase (EC 1.2.4.4), along with *bkdB*, coding for a dihydrolipoyl lysine-residue acetyltransferase (EC 2.3.1.12), and with *lpdA*, encoding a dihydrolipoyl dehydrogenase (EC 1.8.1.4), showed a strong upregulation (FC > 9) in the *phaR* mutant (Table S3). In the proteomic approach, the polypeptides corresponding to BkdA1, BkdA2, and BkdB were present only in the *phaR* mutant, and in the case of the LpdA protein, it showed a higher abundance in the mutant in comparison with the wild type (Table S3). Moreover, another predicted operon related to valine catabolism (blr3954-blr3958) was upregulated (two- to four-fold) in the *phaR* mutant (Table S3). These genes code for a methylmalonate-semialdehyde dehydrogenase (Blr3954), an acyl-CoA dehydrogenase (Blr3955), an enoyl-CoA hydratase (Blr3956), a 3-hydroxyisobutyrate dehydrogenase (Blr3957), and an acyl-coenzyme A synthetase (Blr3958). In turn, the encoded polypeptides were more abundant in the *phaR* mutant, with log_2_ FC values ranging from 2.4 to 5 (Table S3). Together, these enzymes may participate in the degradation of branched-chain amino acids to acetyl-CoA.

Afterwards, the acetyl-CoA generated after oxidation of pyruvate produced in the EMP glycolytic pathway or after branched-chain amino acids degradation may be converted to acetyl-phosphate in a reaction catalyzed by phosphate acetyltransferase (Pta, EC 2.3.1.8) and, in a second step, the high-energy phosphate bond of acetyl-phosphate may serve as phosphate donor to generate ATP from ADP in a coupled reaction catalyzed by acetate kinase (EC 2.7.2.1). Three separate genes encoding this pathway were strongly induced in the *phaR* mutant: *pta* (blr3457) and two *ackA* paralogs (bll2517 and blr3458) (Table S3). We also found correlation in peptides abundances, showing higher levels in *phaR* mutant (Table S3).

Acetyl-CoA may also enter the tricarboxylic acids (TCA) cycle for reducing power building and electrons transfer to oxidative phosphorylation for ATP synthesis. In this sense, polypeptides corresponding to citrate synthase (Blr4839, EC 2.3.3.1), alpha-ketoglutarate dehydrogenase (SucA, EC 1.2.4.2), and succinate dehydrogenases (SdhA and SdhB, EC 1.3.99.1) (Table S3) showed a higher abundance in the *phaR* mutant, indicating that the flux through TCA cycle might be increased. If we assume that the EMP pathway is less efficient than PP/CBB pathways in *B. diazoefficiens* from the point of view of metabolic flux, stimulation of acetyl-phosphate and TCA pathways might compensate for a possible shortage of ATP synthesis rate in the *phaR* mutant.

In parallel with the use of carbon skeletons for the TCA cycle, N from amino acids catabolism should be directed to the urea cycle for secretion. In this regard, the products of blr4358 encoding an N-acetyl-gamma-glutamyl-phosphate reductase (EC 1.2.1.38) and of blr4582 (*argD2*) encoding one of the two annotated acetylornithine/N-succinyldiaminopimelate aminotransferases (EC 2.6.1.11/2.6.1.17) were also more abundant in the *phaR* mutant (Table S3). These enzymes catalyze the third and fourth steps of ornithine production from glutamate. Moreover, ornithine concentration may also be increased due to downregulation of *rocD* (blr3010), which encodes an ornithine aminotransferase (EC 2.6.1.13) that removes ornithine from this pathway towards proline synthesis (Table S3). Increased flux to ornithine might favor citruline synthesis, the next step in the urea cycle, which may react with aspartate to give L-arginosuccinate and then fumarate, or be converted directly to arginine. In this regard, another upregulated gene in the *phaR* mutant, blr4687 (*asd*), encodes one of the two predicted aspartate-semialdehyde dehydrogenases (EC 1.2.1.11) in *B. diazoefficiens*, which catalyzes the transformation of β-aspartyl phosphate to L-aspartate-β-semialdehyde. The possible increase in aspartate-semialdehyde dehydrogenase may prevent aspartate entry into the urea cycle, thus channeling citrulline to arginine, avoiding fumarate loss. Interestingly, although in the *phaR* mutant the NADP-dependent succinate semialdehyde dehydrogenase (EC 1.2.1.16, bll3998, FC = 4.7) is stimulated by FixK_2_ to promote a bypass in the TCA cycle [24], it appears as inversely regulated in proteomic data (log_2_ FC = −2.5) (Tables S1 and S2), indicating a possible regulation at the posttranscriptional level. Taken together, all these reactions might contribute to divert N from amino acids to the urea cycle.

### 2.5. PhaR Showed a Remarkable and Diverse Regulatory Function

In addition to *fixK_2_* and *nifA* regulatory genes, and *rpoN_1_* and *rpoN_2_* sigma factor genes, we found a group of 57 genes coding for transcriptional regulators with a differential expression at transcriptional level in the *phaR* mutant compared to the wild type (21 downregulated and 36 upregulated; Table S1). Among them, we found two of the direct FixK_2_ targets, *nnrR* and *fixK_1_*, that code for CRP/FNR-type transcription factors. The other genes encode regulators belonging to a wide variety of different families such as LysR, MarR, LuxR, TetR, MerR, GntR, RpiR, AraC, IclR, LacI and OmpR-like, that are implied in the control of diverse metabolic pathways. This makes it difficult to assign a role of PhaR in the regulatory network in which they are involved.

Our data also revealed that PhaR may exert a repressive function on chemotaxis and mobility-related genes/proteins (Tables S1 and S2). Specifically, in the *phaR* mutant, the expression of genes encoding four methyl-accepting chemotaxis proteins, three regulators (*cheW*, *cheW*-like, and *cheR1*), and genes related to type IV pilus assembly was induced (two- to four-fold). Similarly, *lafR* (blr6846), which codes for the master regulator (OmpR-like) of lateral flagella in *B. diazoefficiens* [25], was upregulated in the *phaR* mutant (FC = 2.5), but this kind of regulation was not reflected on the expression of the downstream cascade comprising body, hook, or flagellins genes under LafR control. An intriguing exception was *lafA2* (bll6865), which was strongly upregulated (FC = 7.2) in the *phaR* mutant and, remarkably, it is the only lateral flagellar gene not controlled by LafR [25]. These results suggest that PhaR may be a master regulator of both *lafR* and *lafA2* genes.

In contrast to lateral flagella, we could observe that all the four flagellins of the constitutively expressed *B. diazoefficiens* subpolar flagellum [26]; Bll5843-46 (FliC1-4) and a flagellar hook protein (Bll5854) were more abundant in the *phaR* mutant than in wild-type cells (Table S2), in accordance with upregulation of their master regulator *ctrA* (blr2200) with an FC= 2.2 (Table S1).

### 2.6. Other Genes/Proteins Are Controlled by PhaR

In addition to the described genes/proteins under PhaR control, we observed that bll5770 (FC = 3.2) coding for the alkyl hydroperoxide reductase AhpD, bll6069 (FC = 4.8) and bll2590 (FC = 5.1), both coding for universal stress protein A, were upregulated in the *phaR* mutant (Table S1). PhaR mutation also led to an increased abundance of peptides of eight aminoacyl-tRNA ligases/transferases (Table S2). These data suggested that the absence of PhaR induces some sort of stress response, usually mediated by reactive oxygen species [27,28].

Finally, mutation in *phaR* also affected the expression of 121 genes coding for putative ABC transporters, with 107 of them being downregulated. In addition, 243 genes coding for hypothetical/unknown proteins were also affected (Table S1). This latter group constitutes about 20% of the total number of differentially expressed genes in the *phaR* mutant, which points out that PhaR might be involved in metabolic processes that remain to be discovered.

### 2.7. Identification of PhaR Direct Targets

Next, in order to identify genes directly controlled by PhaR, we sought, in a first step, to define this protein core regulon. For this purpose, the focus was set on the overlap between transcriptomic and proteomic datasets, and, specifically, on those targets that showed the same profile of control (up- or downregulation) at the transcriptional and protein levels in the *phaR* mutant compared to the wild type. These analyses allowed us to select two groups of targets: one comprising 45 genes with induced expression in the *phaR* mutant (i.e., repressed by PhaR), organized into 29 putative transcriptional units (Table 1); the second containing 108 genes with reduced expression (i.e., activated by PhaR), organized into 78 putative transcriptional units (Table S4).

Since PhaR is mainly recognized as a repressor [7,12,18], for the prediction of a PhaR-binding site, we focused on the targets with induced expression in the *phaR* mutant. In these in silico analyses, we also considered a genuine PhaR target, *phaP1* (bll5155) [18], despite that it is not present in our selected transcriptomic/proteomic overlapping dataset. This analysis yielded a total of 30 putative promoters to search for a consensus PhaR motif (Table 1).

**Figure 5:**
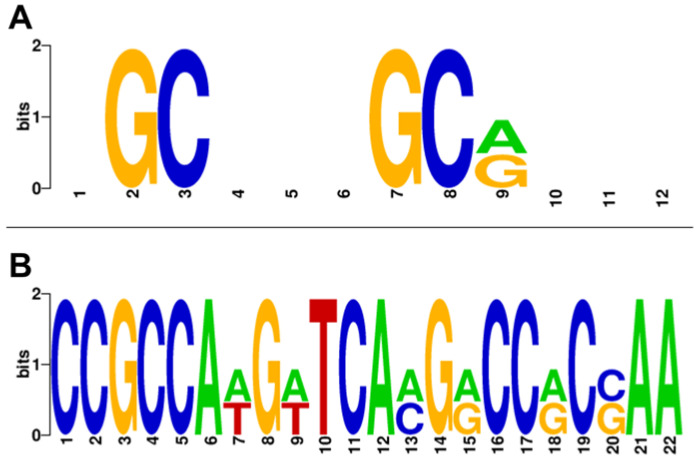
Prediction of putative PhaR–DNA-binding sites. (**A**) Discovered PhaR consensus binding site. The motif NGCN(3)GC[GA]N(3) is 12 pb long and enriched in GC. (**B**) Alternative novel PhaR gapped binding site. The motif CCGCCA[AT]G[AT]TCA[CA]G[GA]CC[GA]C[GC]AA is 22 bp long, also enriched in GC, and preferentially found in repressed genes. The logos were created using the “WebLogo” tool (https://weblogo.berkeley.edu/logo.cgi; accessed on 27 November 2023) [29]. The colors represent the different nucleotides.

Predictions for a PhaR-binding site based on the MEME suite (https://meme-suite.org/meme/; accessed on 18 May 2023) unveiled a series of motifs with a similar pattern enriched in guanine and cytosine. Following these findings and taking into consideration the PhaR-binding site described by Nishihata and coworkers [18], a consensus sequence of 12 nucleotides was defined, i.e., NGCN(3)GC[GA]N(3) (Figure 5A; N is any nucleotide [A, T, C or G]) that were present in 25 out of the 30 promoters used for the prediction (approximately 83%), as a single or multiple location (Table 1).

Interestingly, this consensus sequence was also found in 66 out of the 78 promoters associated with genes with downregulated expression in the *phaR* mutant (approximately 85%), also as single or multiple presence (Table S4). Based on these observations, we would conclude that PhaR could bind to a similar DNA sequence (PhaR box) for both repression and activation of gene expression.

Among the five promoters associated with PhaR-repressed genes that did not harbor this 12 bp regular pattern within their respective promoter regions (about 17% of the set), we found two out of the top ten upregulated genes in the *phaR* mutant (Table 1). These comprised blr4358 with an FC = 8.7, and blr4687 (*asd*) with an FC = 9.1. A search for motifs with variable gap length in order to find a possible alternative repression motif (GLAM2, https://meme-suite.org/meme/; accessed on 6 June 2023 [30]) unveiled a longer alternative pattern of 22 nucleotides present in these two promoters (Table 1). The novel pattern was also enriched in G and C, i.e., CCGCCA[AT]G[AT]TCA[CA]G[GA]CC[GA]C[GC]AA (Figure 5B), that will be referred to as “long PhaR box”. This outcome supports the existence of alternative PhaR-binding sites with different sequence motifs containing longer or shorter gaps. However, this recognition site seemed to be exclusive for the selected PhaR-repressed genes, as it was not found in the group of the 78 PhaR-activated gene promoters (Table S4).

**Figure 6:**
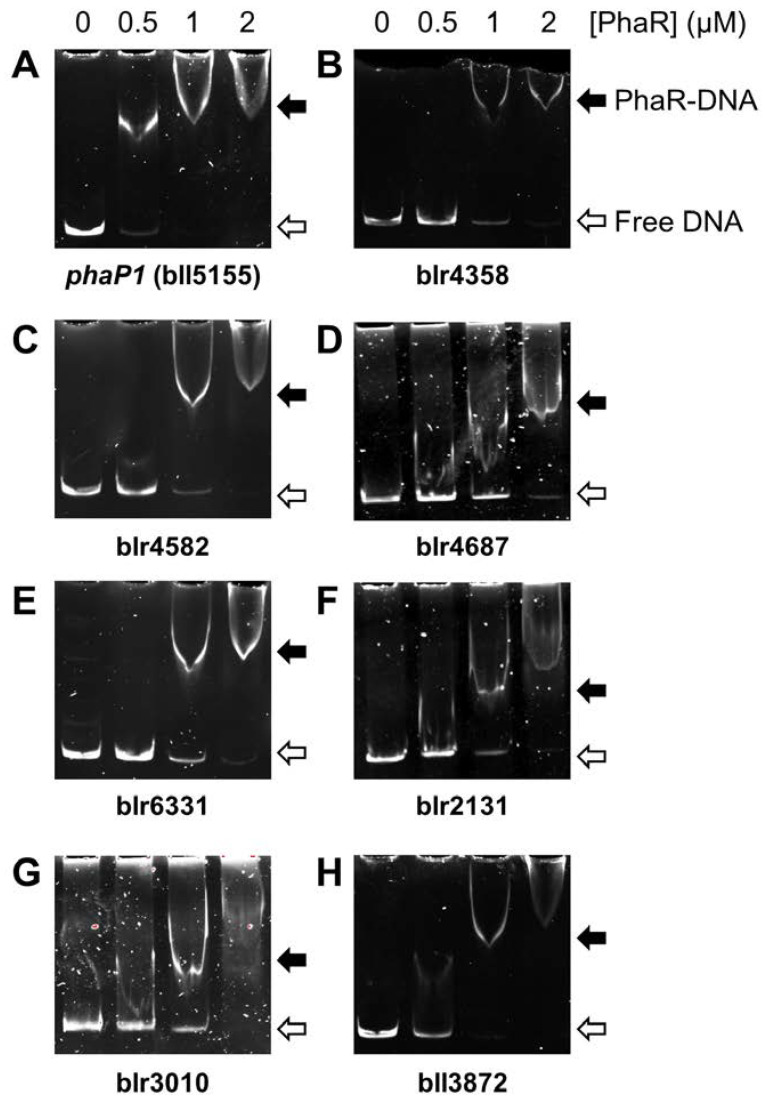
PhaR interaction with promoter regions of a selection of PhaR-regulated genes using EMSAs. Purified recombinant PhaR protein at increasing concentrations (0, 0.5, 1, and 2 µM, indicated at the top of each set of gels) was incubated with 2 ng of individual PCR products comprising the promoter regions of five PhaR-repressed genes: bll5155 (*phaP1*), blr4358, blr4582, blr4687, and blr6331 (**A**–**E**), and of three PhaR-activated genes: blr2131, blr3010, and bll3872 (**F**–**H**). Black arrows, PhaR–DNA complexes; white arrows, free DNA.

Direct interaction of purified PhaR with a selection of the top ten candidates, either repressed (blr4358, blr4582, blr4687, blr6331) or activated by PhaR (blr2131, blr3010, bll3872), was next verified by DNA electrophoretic mobility shift assays (EMSAs) (Figure 6). The promoter region of the genuine PhaR target *phaP1* (bll5155) was also included in the experiments. The target DNA sequences for these experiments were generated by PCR amplification of the promoter regions as ~300–400 bp fragments using suitable primers (Table S5). We found that, in the case of *phaP1*, PhaR–DNA interaction was readily detected when 0.5 μM PhaR protein was included in the reaction (Figure 6, panel A). However, the binding of purified PhaR protein to the other seven target promoters was only observed at a concentration at or above 1 µM PhaR (Figure 6, panels B–H). This points out that blr2131, blr3010, bll3872, blr4358, blr4582, blr4687, and blr6331 are seven new putative direct targets of PhaR, but the affinity of PhaR for these promoters is lower than for the promoter of the *phaP1* gene.

### 2.8. Functional Mutagenesis of the phaP1 Promoter

**Figure 7:**
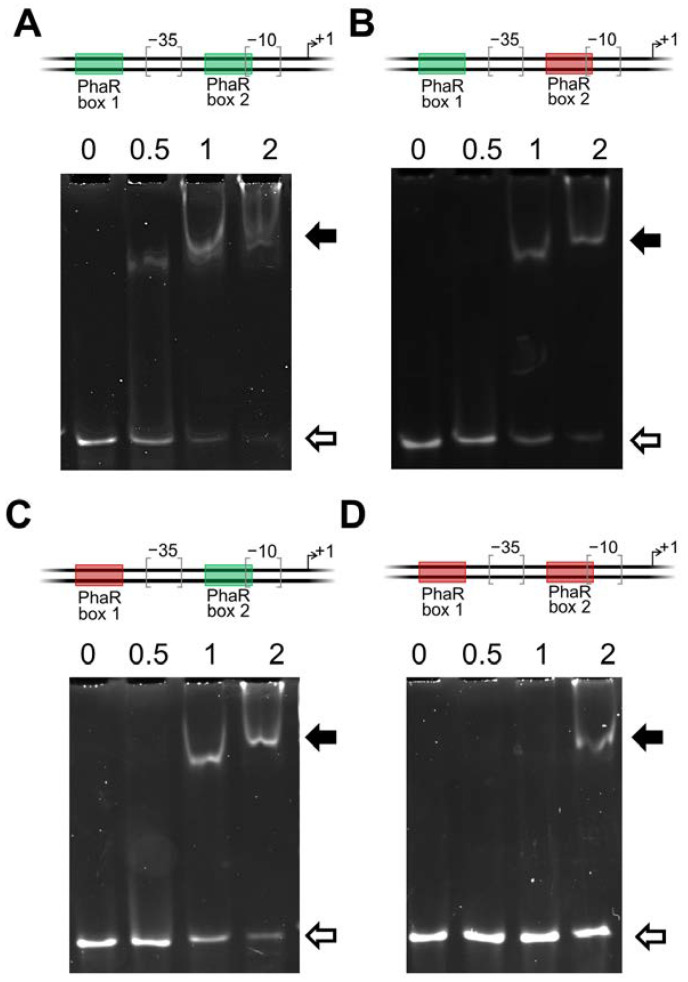
Functional mutagenesis of PhaR boxes present within the *phaP1* promoter. The −10 and −35 regions and the transcription start site of *phaP1* are depicted at the top of each panel (**A**–**D**). The two predicted PhaR-binding sites are symbolized with boxes: green, parental box; red, mutated box (transversion of G residues to T residues and C residues to A residues at positions 2, 3, 7, and 8 of the box). Analysis of PhaR interaction with the four *phaP1* promoter variants was tested by EMSAs. Synthetic double-stranded oligonucleotides containing the two genuine PhaR boxes (**A**), with a mutation in the distal (**B**) or in the proximal box (**C**), and with a mutation at both boxes (**D**), were mixed with increasing concentrations of recombinant purified PhaR protein (0, 0.5, 1, and 2 µM, indicated at the top of each gel). PhaR–DNA interaction was determined by protein–DNA complexes formation (black arrow) or free DNA disappearance (white arrows).

As observed for some other putative PhaR targets (Table 1 and Table S4), the promoter of *phaP1* (bll5155) harbors two PhaR-binding sites (Figure 7). Notably, they are separated by 17 nucleotides, flanking the −35 region of the *phaP1* promoter and, in fact, the proximal site actually overlaps with the −10 region. Although it has been reported that PhaR can bind to these two 12 bp regular patterns simultaneously [18], a deeper study to uncover the relevance of each binding site for PhaR interaction has not yet been performed.

Within the 12 bp regular pattern N**GC**N(3)**GC**[GA]N(3), two contiguous GC sequences at positions 2, 3, 7, and 8 (in bold) that are separated by three nucleotides are conserved. In order to test the functionality of either of the two PhaR boxes associated with *phaP1* promoter and, in particular, the putative role of the tandem GC nucleotide sequences of each binding site, a battery of double-stranded oligonucleotides of 67 bp was prepared (Table S5). Both boxes were individually or simultaneously mutated by transversion of G residues to T residues, and C residues to A residues at positions 2, 3, 7, and 8 of each of the PhaR boxes, yielding four *phaP1* promoter variants: the parental harboring the two genuine PhaR-binding sites, one with a mutated proximal site, one with a mutated distal site, and the fourth with both sites mutated (Figure 7).

As observed with the PCR product containing the *phaP1* promoter (Figure 6), an effective binding of PhaR to the wild-type double-stranded oligonucleotide was already observed at a concentration of 0.5 µM of protein (Figure 7, panel A). However, a concentration of at least 1 μM was required to detect PhaR binding to the *phaP1* promoter with mutated distal or proximal PhaR box (Figure 7, panels B and C). Furthermore, a weak binding was observed when both PhaR boxes were mutated, even when four-fold protein concentration (2 µM) was added to the reaction (Figure 7, panel D). This is an indication that both boxes are important for PhaR binding and, indeed, that the double GC sequence in tandem plays a key role in this interaction.

### 2.9. PhaR Binds to DNA as a Multimeric Form

**Figure 8:**
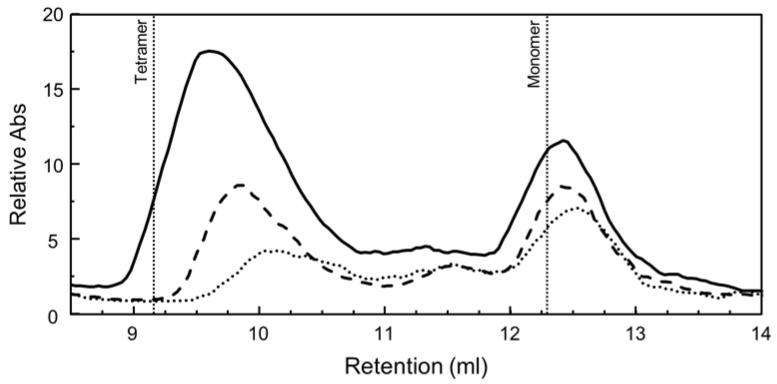
Determination of PhaR oligomeric state by SEC. A total of 200 µL of untagged PhaR protein sample at 30, 15, and 10 µM (solid, dashed, and dotted lines, respectively) were loaded onto a Superdex 75 10/30 GL column and separated at an isocratic flow of 0.5 mL/min. Elution profiles monitored at 220 nm showed two peaks corresponding to the tetrameric and monomeric forms of PhaR. The vertical lines show the calculated elution volume of the theoretical Mw of the monomer (~22.6 kDa) and the tetramer (~90 kDa) of PhaR. Absorbance data were normalized to blue dextran values for each run.

The functional mutagenesis of the *phaP1* promoter suggested that *B. diazoefficiens* PhaR could bind DNA as a multimeric form. Therefore, to determine the solution oligomeric state of PhaR, size-exclusion chromatography (SEC) experiments of recombinant untagged PhaR of *B. diazoefficiens* were carried out (Figure 8). Each PhaR protein preparation, prior to SEC experiments, was analyzed by denaturing sodium dodecyl sulfate polyacrylamide gel electrophoresis (SDS-PAGE) [31], showing a purity greater than ~95% for the band corresponding to the predicted molecular mass of PhaR (~22.6 kDa) (Figure S1; a standard protein purification profile is shown). The chromatographic profile of PhaR at a concentration of 30 µM indicated that it eluted predominantly as tetramer (apparent molecular weight of about 83,000). However, at lower concentrations (15 and 20 μM), the second peak of an apparent molecular mass of about 22,000, corresponding to the monomer, increased. These results suggest that, although PhaR behaved mainly as a tetramer in solution, some fraction of this form dissociated during elution, which increased at low protein concentrations. Interestingly, the PhaR ortholog of *Paracoccus denitrificans* also forms a homotetramer in the native state [7,32].

### 2.10. Identification of PHB Granule-Associated Proteins in Wild Type Cells

PhaR possesses not only a DNA-binding domain but also a PHB accumulation regulatory domain. Therefore, we wanted to analyze whether PhaR is able to bind to PHB granules in *B. diazoefficiens* cells. For that purpose, we extracted PHB granules from wild-type cells cultured under our assayed conditions (microoxia with mannitol as a carbon source), and the associated proteins to PHB granules were isolated and identified by using liquid chromatography–tandem mass spectrometry (LC–MS/MS) in combination with electrospray ionization-ion trap (ESI-IT).

We found a group of 427 proteins associated with PHB granules. This large set of proteins presumably includes false-positive due to random binding to the hydrophobic PHB surface after cell lysis [33] and/or binding of PHB to released phospholipids during the granule isolation process [34,35]. Therefore, we focused on those proteins putatively related to PHB metabolism in *B. diazoefficiens*. Of these proteins, we found seven with a score > 50 and two or more cryptic peptides per protein (Table S6). As expected, we identified PhaR, PhaA2, and PhaB2 proteins, but we did not find either PhaC or PhaZ paralogs. Interestingly, while phasin-encoding genes *phaP1*, *phaP2*, *phaP4,* and *phaP5* had the highest levels of expression at transcriptional level in the wild-type strain, only PhaP1, PhaP2, and PhaP3 proteins were found attached to PHB granules. In turn, PhaP2 was the only phasin that was overexpressed in wild type compared to *phaR* mutant, and PhaR seems to exert no control on this paralog. Unexpectedly, we cannot find PhaP4 attached to PHB granules, the phasin with highest mRNA signals in the wild type (Figure 3) and with the highest affinity to PHB in vitro [5].

We also identified acetyl CoA synthase (Blr3924) and acyl-CoA synthetase (Bll2619), enzymes that synthesize acetyl-CoA from acetate. These enzymes were localized previously in PHB granules of *Pseudomonas putida* [36,37].

As described for other bacterial species [38], other proteins with no relation to PHB metabolism were also found associated with PHB granules in this study (Table S6), for instance, phosphoenolpyruvate carboxykinase (Bll8141, PckA), ABC transporters (Bll6453), pyruvate dehydrogenase E1 component β subunit (Bll4782, PdhB), pyruvate dehydrogenase E1 component α subunit (Bll4783, PdhA), and acetyltransferase component of pyruvate dehydrogenase complex (Bll4779) [34]. Whether these proteins specifically bind to PHB has not yet been experimentally proved.

We also identified nine and five peptides unique to AcrA (Blr1515 and Bll3872, respectively) and eight peptides unique to AcrB (Bll3871) proteins. Two of these three genes that encode integral membrane proteins belonging to efflux RND transporter system were the most strongly activated by PhaR in our microarray data (FC −2.6, −55.1, and −72.8, respectively; Table S1). We do not know whether these three proteins bind specifically to the surface layer of PHB granule to transport substrates or if their finding associated with PHB might be due to their hydrophobic/amphiphilic properties. It is worth mentioning that the genome of *B. diazoefficiens* USDA 110 [4] encodes 24 members of the family of RND multidrug efflux systems [39]. More studies are needed to elucidate if this AcrAB system plays a specific role in PHB metabolism.

## 3. Discussion

The present study reports experimental evidence that *B. diazoefficiens* PhaR is involved in genetic and metabolic control in free-living cells growing under oxygen-depleted environments. Our multidisciplinary approach covering transcriptomics, proteomics, bioinformatics, molecular biology, and biochemical techniques has expanded our understanding about the function of PhaR and its associated molecular mechanism, revealing a pleiotropic role for this protein that includes not only regulation of the PHB cycle and the microaerobic metabolism in *B. diazoefficiens*, but also an involvement in global carbon and nitrogen allocation, bacterial physiology and motility, and stress response.

First, as observed in many other bacterial species, PhaR regulates PHB accumulation by modulating (repressing) genes related to PHB synthesis. In addition, the absence of PhaR led to a growth deficiency ([2], this study), in which PHB synthesis is arrested. We showed that, even when *phaA2* and *phaB2* levels were increased in the *phaR* mutant (Table S1), their protein abundances were similar to the wild-type strain (Table S2). However, the presence of PHB depolymerase PhaZ1 only in the *phaR* mutant strongly suggests that PHB granules are subject to degradation. As previously demonstrated, the *phaR* mutant did not produce PHB during stationary phase under the same culture conditions employed in our study [2], suggesting that either the ratio of acetyl-CoA/acetate remains low and/or availability of carbon backbones and reducing power in these cells is insufficient.

Based on our transcriptomic data, PhaR seems to repress both *phaC2* and *phaC5* genes (although the latter is expressed at very low levels). If we assume that these genes do not encode active PHB synthases [1], the only active PHB synthase (PhaC1) was not controlled by PhaR. Therefore, the reduced PHB content in the *phaR* mutant cells (Figure 1) can be explained based on the following observations: (i) higher PHB degradation by overexpression of *phaZ1*/PhaZ1 ([40], this work), (ii) higher expression levels of *phaC2* and *phaC5* that do not code active PHB synthases ([1], this work), and/or (iii) formation of less-active heterodimers PhaC1/PhaC2 rather than fully active homodimers PhaC1/PhaC1, due to higher *phaC2* transcription and a constant transcription of *phaC1* ([1,2,22,41], this work) (Figure 3B). In addition, since PHB is not synthesized, PhaR would be free to bind to DNA and exert its activity as a transcriptional regulator rather than PHB modulator. Moreover, in the wild type growing with mannitol as the sole carbon source at high C/N ratio, an excess of reducing power may be produced. In this scenario, PhaR could restructure carbon metabolism to direct reducing power towards CBB cycle [42,43,44] and the consequent excess of NAD(P)H building towards PHB synthesis, and at the same time, avoid loss of amino acids for ATP production.

The role of phasins in *B. diazoefficiens* microaerobic metabolism seemed to be more intriguing. Five out of the seven predicted phasins, i.e., PhaP1, PhaP2, PhaP3, PhaP4, and PhaP5, were detected in both the wild-type and the *phaR* mutant strains. These five proteins also correspond to their highest transcript levels observed in the microarray data (Figure 3C). But only three of them, PhaP1, PhaP2, and PhaP3, were found to be associated with PHB granules. Unexpectedly, we did not find PhaP4 attached to PHB granules extracted from *B. diazoefficiens* cells, despite its highest transcriptional levels and highest affinity for PHB in vitro [5]. We also found that PhaR did not control *phaP2* transcription (Figure 3B), and at the same time, PhaP2 abundance was higher in the wild-type strain. Considering that this phasin paralog was poorly expressed in oxic cultures [5], a role of this phasin in PHB microoxic metabolism became evident. However, phasins can play different roles regardless of their function on PHB granules [14,45]. Thus, the precise function of each phasin paralog in *B. diazoefficiens* metabolism remains enigmatic.

Moreover, the presence of PhaR adhered to the granules and the absence of PhaZ1 at the time of harvest (mid-exponential phase) in the wild-type strain suggest that there is little or no PHB degradation, consistent with the observed PHB accumulation, possibly due to an excess of reducing power, without turnover, as suggested in reference [46]. However, as we could not identify any PHB synthase peptides by proteomics, we can only speculate about PHB dynamics under our experimental conditions.

In addition to PHB metabolism control, mutation of *phaR* induced alterations in carbon flux through metabolic pathways. We provide evidence that four genes/enzymes of the TCA cycle and three key genes/enzymes of acetate metabolism are increased in the *phaR* mutant compared to the wild type (Tables S1 and S3). Thus, acetyl-CoA (probably synthesized from EMP glycolytic pathway and/or after branched-chain amino acids degradation) was diverted into the TCA cycle and the acetate kinase (Ack)-phosphate acetyltransferase (Pta) pathway towards ATP synthesis. If we assume that the EMP pathway is less efficient than PP/CBB pathways in *B. diazoefficiens* from the point of view of metabolic flux, the stimulation of the acetyl-P and TCA pathways could compensate for a possible shortage of ATP synthesis rate in the *phaR* mutant. Moreover, acetate overflow controls acetyl-P levels in bacteria [47], which, in turn, can directly phosphorylate and activate several bacterial response regulator proteins [48].

Three individual pieces of evidence (transcriptomic and proteomic assays and protein–DNA-binding studies) suggested that PhaR is involved in the control of amino acid metabolism under microaerobic conditions. Interestingly, the four new direct PhaR targets subjected to a negative control are involved in amino acids synthesis (blr4358, blr4582, blr4687 [*asd*], blr6331[*bkdA1*]). In addition to the repression in the pathway of lysine synthesis from aspartate, the production of diaminopimelate, an intermediate towards lysine, might be compromised. Diaminopimelate plays an essential role in bacterial cell wall formation. Indeed, *asd* mutation resulted to be lethal for *Salmonella typhimurium* [49] and *Legionella pneumophila* [50]. However, to our knowledge, no functional studies on *asd* have been reported so far in rhizobia.

PhaR also functions as an activator in amino acid catabolism. Two positively controlled PhaR genes, i.e., blr3010 and blr2131, showed a downregulated expression in the *phaR* mutant. The product of the first is an ornithine aminotransferase (EC:2.6.1.13) implicated in arginine and proline degradation. The blr2131 gene is part of the predicted blr2131-blr2136 operon and encodes a lysine N6-hydroxylase (EC:1.14.13.59) which might have a role in lysine degradation according to in silico predictions. The positive control of PhaR over these two targets implied in arginine and lysine degradation is coherent with the repression of genes involved in their biosynthesis (see above), indicating that PhaR could favor amino acid turnover.

The absence of PhaR provoked arrested PHB synthesis, imbalance of growth rate, and strong carbon allocation under microaerobic conditions, suggesting the existence of low ATP/ADP, NADH/NAD^+^, and/or acetyl-CoA/CoA ratios in the *phaR* mutant. In this regard, we found that *hup*, ferredoxin/flavoproteins-like, and cytochrome-related genes, which are important for hydrogen recycling, respiratory chain, and electron transfer reactions, respectively, were downregulated in the *phaR* mutant (Table S1). Based on these observations, we can hypothesize that few protons (H^+^) are diverted to ATP production when PhaR is absent. Taken together, this global regulator appears to play a role in partitioning carbon flux through PP/CBB instead of EMP pathways, favoring carbon to be catabolized by PP/CBB.

Additionally, our data suggest that either PhaR or PHB accumulation, or both, influences motility by repressing genes related to chemotaxis response regulators, methyl-accepting chemotaxis proteins, and master regulators encoding both (subpolar and lateral) flagellar systems (Table S1) in *B. diazoefficiens* [51]. Since all four flagellins of the subpolar flagellum were detected in the proteomic analysis, we speculate that this flagellum is primarily responsible for motility in our microaerobic conditions and it might be also present in flooded soils, where this bacterium can live. This is consistent with observations of increased competitiveness for nodulation by *lafA* mutants in such environments [52]. Additionally, the master regulator of lateral flagella, *lafR*, which is necessary for the expression of the lateral flagellar system, was found to be repressed by PhaR (Table S1). However, all lateral flagellar genes were poorly expressed in both wild-type and *phaR* mutant cells, and, indeed, none of the approximately 70 lateral flagellar proteins were detected in the proteomic approach ([25], this work). There is not a straightforward reason why *lafR* transcripts are not correlated with the downstream cascade for flagellar formation, which will be interesting to pursue in future studies. Evidence for a role of PhaR in motility and PHB accumulation was already shown in previous studies. This is the case for *B. diazoefficiens* [18] as well as for *Xanthomonas oryzae* (a rice pathogen), where a *phaR* mutant displayed a hypermotile swimming phenotype on agar plates [53]. Other studies linked PHB accumulation and flagellar or pilus-mediated motility in other Gram-negative bacteria, such as *Azospirillum brasilense*, *Ralstonia eutropha* [54,55,56], and *Pseudomonas extremaustralis* [57], based on the phenotype of *phaC* mutant cells that contains reduced PHB levels under cold conditions. Overall, it seems that the accumulation of PHB is linked to the flagellation state, regardless of the bacterial species.

**Figure 9:**
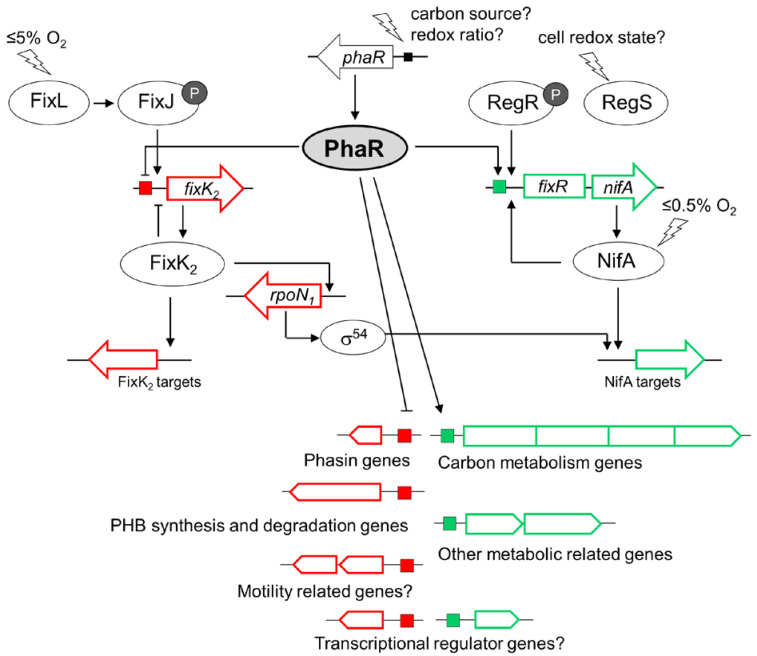
PhaR acts as a modulator of the FixLJ-FixK_2_ and RegSR-NifA microoxic-responsive regulatory network in the *B. diazoefficiens* free-living lifestyle. Genes are depicted by boxes and open arrows, and proteins by ovals. Positive regulation is denoted by arrows, and negative regulation is indicated by perpendicular lines. Boxes at the gene promoters represent recognition sites of PhaR as a repressor (red) or activator (green). PhaR-repressed and activated genes are colored in red and green, respectively. Signals are represented by lightning bolts. The phosphoryl groups of FixJ and RegR response regulators are depicted by small black circles labeled as “P”. The signal(s) for activation of *phaR* expression are unknown.

Remarkably, our transcriptomic/proteomic data also expanded the regulatory function of PhaR and revealed a pivotal control on FixK_2_- and NifA-dependent regulons, repressing the former and activating the latter, unveiling a role of the PhaR protein as a modulator of both cascades that respond to different oxygen thresholds within the microaerobic regulatory network in *B. diazoefficiens* (Figure 9) [58]. Interestingly, PhaR was able to interact individually with both *fixK_2_* and *fixR*-*nifA* promoter regions (Figure S2). This finding points out a direct control mediated by PhaR that could be linked to the presence of a 12 bp regular pattern (at position −38) within the *fixR-nifA* operon promoter, but the exact mechanism remains to be investigated. However, in the case of PhaR-*fixK_2_* interaction, neither of the two defined PhaR-binding sites (“short” and “long” PhaR boxes; Figure 5) could be identified within its promoter region, although several GCN(3)GC motifs in tandem resulted to be present. This indicates that additional GC-enriched sites for PhaR might exist.

It is noteworthy that, although the expression of the *fixR*-*nifA* operon and a substantial number of genes belonging to the NifA regulon are downregulated in the *phaR* mutant in cells cultured microaerobic free-living conditions, this mutant exhibited a better symbiotic phenotype than the wild type in soybeans [2]. This is intriguing, because *B. diazoefficiens* mutants in *nifA* and/or *nifD*, *nifK*, *nifH*, *nifE*, *nifN*, *nifS*, *nifB*, *fixA*, *fixB*, or *fixC* genes displayed ineffective soybean nodules [59]. Moreover, peptides of FixR, NifH, and NifK proteins were only found in wild-type cells (Table S2). One plausible explanation for this apparently controversial phenotype could be as follows: although under microaerobic free-living conditions the expression of *regR* was not affected in the *phaR* mutant (Figure 4A), RegR activation (i.e., phosphorylation) could be impaired, resulting in a lower ATP/ADP ratio. In turn, *fixR*-*nifA* and the downstream controlled genes are downregulated with direct consequences on nitrogen fixation and hydrogen uptake processes, which require high levels of ATP [60].

In this work, we also identified seven new direct targets for PhaR which are involved in amino acids metabolism or detoxification processes, among others (see above), highlighting the pleiotropic role of this regulator. The comparative analysis of transcriptomic and proteomic data helped the definition of a core regulon that served as a basis for the prediction of a DNA consensus sequence for PhaR, which facilitated the identification of direct targets by using protein–DNA-binding studies. It is worth mentioning that Maehara and coworkers [7] proposed the possibility of a conserved binding site for PhaR regulators (a so-called “PhaR box”). In particular, in *P. denitrificans* or *Rhodobacter sphaeroides* 2.4.1, PhaR recognizes a common motif that includes repeated TGC sequences (namely, TGC-rich region) [7]. Furthermore, the number of these TGC-rich regions could determine the PhaR-binding affinity for DNA.

In our bioinformatics approach, two binding motifs for *B. diazoefficiens* PhaR were predicted (Figure 5). The first motif is a 12 bp regular pattern containing a conserved GCN(3)GC sequence, present in different number and at distinct distribution within promoters (Figure 5A; Table 1 and Table S4). However, as the GC sequence in tandem is presumably a quite-abundant motif in *B. diazoefficiens* genome, we believed that its redundancy within the promoter region, the distance from the transcriptional start site, and the possible interaction with other regulator/s might contribute to PhaR-mediated control. In fact, PhaR bound with a higher affinity when the regular pattern was located in tandem, and relatively close each other, as was the case of the *phaP1* promoter, where protein–DNA interaction was already observed at 0.5 μM of protein (Figure 6). Indeed, within this promoter, PhaR not only bound, probably as a tetramer (Figure 8), to both the distal and the proximal sites, but, also, both sites seemed to be required for full PhaR interaction (Figure 6 and Figure 7). This is in line with the findings by Nishihata and coworkers that reported that PhaR is able to bind to both sites based on DNA I footprinting assays [18]. Here, we identified the two GC sequences in tandem of each site as a crucial element for protein–DNA interaction (Figure 7).

The second, novel, alternative, and longer 22 bp pattern for PhaR (“long PhaR box”) was identified within the promoter region of two repressed genes, i.e., blr4358 and blr4687, which is also enriched in G and C (Table 1). The effective binding of PhaR to this longer sequence was also confirmed in our study (Figure S3); however, a functional mutagenesis at the invariable G and C positions of the motif present at the blr4358 promoter, similar to that of the one performed with the *phaP1* promoter (transversion of G versus T residues and C versus A residues), did not affect PhaR–DNA interaction in the range of 1–2 μM of protein (Figure S3).

Similar functional mutagenesis was also performed at the blr2131 promoter, a target activated by PhaR which harbors three regular patterns starting at position −152, −138, and −105 from the predicted start codon (Figure S3 and Table S4). This approach unveiled that, in this particular case, the two more proximal boxes were functional, since a higher concentration of PhaR, 2 μM instead of 1 μM, was required to detect an effective interaction of the protein with the mutant promoters, but the effect was less radical than that observed in the *phaP1* promoter (Figure S3, panels C and D versus Figure 7). However, a milder effect on PhaR–DNA-binding was detected when mutations in the most distal PhaR box were introduced (Figure S3, panel B), indicating that it residually contributes to PhaR binding.

In summary, the results of this work provide supporting evidence that PhaR is a transcription factor with multiple purposes in *B. diazoefficiens*, acting as a negative regulator of PHB cycle, as in other bacteria, but also as a positive and negative modulator of the global metabolic network in microaerobic environments. PhaR function implies binding to two different target biomolecules: fatty acid (PHB granules) and DNA. Their targets are genes and proteins involved in PHB synthesis and degradation, genes that encode diverse transcriptional factors, as well as genes related with motility and key central carbon and nitrogen metabolic pathways, which reinforce its pleiotropic role.

## 4. Materials and Methods

### 4.1. Bacterial Strains and Culture Conditions

Strains are summarized in Table S7. *B. diazofficiens* strains were grown at 30 °C, while *E. coli* strains were grown at 37 °C. The wild-type *B. diazoefficiens* strain used in this work (LP 3004) is a spontaneous Sm-resistant derivative from the type strain *B. diazoefficiens* USDA 110 and the *phaR* mutant strain is a LP 3004 insertional mutant derivative, in which the plasmid pIQ36 was inserted in the middle of the *phaR* coding region [2]. For PHB extraction, microarray, and total protein extraction experiments, *B. diazoefficiens* strains were first grown oxically in PSY-arabinose cultures with appropriate antibiotics for 4–5 days (50 mL/250 mL) [17,61]. The cultures were then centrifuged for 10 min at 7000 rpm, and the cell pellets were washed twice with Götz minimal medium without a carbon source. The washed pellets were resuspended in 5–10 mL of the same medium and used to inoculate 40 mL of Götz minimal medium with mannitol in 500 mL flasks at an optical density at 600 nm (OD_600_) of approximately 0.02. Each set of cultures (10 for the wild type and 20 for the mutant strain) was incubated under microaerobic conditions (0.5% O_2_ and 99.5% N_2_ in the gas phase) in rubber septa flasks. The gas phase was exchanged every 8 to 16 h. Total biomass was estimated by the number of viable bacteria by colony-forming units (CFU) counts on YM agar plates [62] and by OD_600_.

### 4.2. PHB Extraction and Quantification

PHB was extracted from washed pellets of wild-type and *phaR* mutant strains cultured microoxically. Then, sodium hypochlorite was added and the mix was incubated overnight at room temperature, precipitated with 1:1 alcohol–acetone, and resuspended in chloroform. Then, each pellet was homogenized with sodium hypochlorite at room temperature, washed with mQ water, precipitated with 1:1 alcohol–acetone, and resuspended in chloroform. PHB was quantified by the spectrophotometric method of Law and Slepecky [63] using commercial PHB as standard (Sigma-Aldrich, Saint, Louis, MO, USA).

### 4.3. Microarray Experiments and Data Analyses

For microarray experiments, a minimum of three independent biological sets per strain were prepared. Cultures were harvested at mid-exponential phase (OD_600_ = 0.25–0.45) by mixing with 0.1 volume of “stop solution” (10% phenol, pH 8, in ethanol), and centrifuged for 10 min (12,000× *g*, 4 °C), as previously described [17,20,64]. Cell pellets were stored at −80 °C or used directly for total RNA isolation [17,20,64]. RNA quality control, cDNA synthesis, fragmentation, labeling, and conditions for hybridization with a custom-designed *B. diazoefficiens* Gene Chip BJAETHa520090 (Affymetrix, Santa Clara, CA, USA) were carried out as described elsewhere [17,20,64,65]. Specifically, 1.8 µg of labeled fragmented cDNA were hybridized to the arrays.

Data analyses were performed as described by Parejo and coworkers [65]. Briefly, signal intensities detection, normalization, and analyses were performed with Affymetrix Expression Console software version 1.4.1 (Affymetrix, Santa Clara, CA, USA). Comparative analyses were performed with Transcriptome Analysis Console 3.1 software (Affymetrix, Santa Clara, CA, USA). Normalized intensities (MAS 5.0 algorithm) were compared between conditions using one-way between-subject ANOVA (ANOVA *p*-value < 0.05). Genes considered as differentially expressed were those that passed the statistical tests and when the change in expression (measured as *n*-fold change [FC] was ≥2 or ≤−2 in comparisons between two strains.

### 4.4. PhaR–DNA-Binding Site Prediction

The workflow of the sequential steps for bioinformatic prediction of putative PhaR-binding sites was as follows. Genes were first grouped into putative operons, as described previously [17,20]. Our own developed R scripts were used to extract 300 bp upstream of each putative promoter from the genomic sequence of *B. diazoefficiens* USDA 110 genome (GCF_000011365.1_ASM1136v1_genomic.fna; https://www.ncbi.nlm.nih.gov/; accessed on 31 August 2016). The resulting BED file and the original GFF annotation file (GCF_000011365.1_ASM1136v1_genomic.gff accessed on 23 November 2016) were used as input for bedtools 2.31.0 [66]. The resulting FASTA file contained all selected promoter regions. PhaR-binding consensus motif sequences were predicted using the generated promoter fasta sequences and the MEME Suite version 5.5.2 (https://meme-suite.org/meme/; accessed on 18 May 2023; [67]. The discovery of PhaR-binding motifs was pursued using different strategies: (i) enrichment of known motifs with SEA tool (default parameters, databases: CollecTF and Jaspar Core 2022); (ii) de novo discovery of motifs without gaps using probabilistic methods with MEME (maximum and minimum motif length, set to 16 and 4 bp, respectively, distribution types Zoops and Anr, rest of parameter as supplied by default); (iii) discovery of fixed-length and gapless motifs using Streme (the maximum and minimum length of columns to align, set to 16 and 4 bp, respectively, distribution types Zoops and Anr, databases: CollecTF and Jaspar Core 2022); and (iv) discovery of motifs with variable gap length with GLAM2 using default parameters. The search of the predicted pattern within the promoter region of genes repressed and activated by PhaR was performed using the *fuzzpro* tool in the EMBOSS suite (https://www.bioinformatics.nl/cgi-bin/emboss/; accessed on 26 May 2023; [68].

### 4.5. Total Protein Isolation, Identification, and Quantification

For proteomic analysis, three independent biological sets per strain were prepared. Cultures were harvested at mid-exponential phase (OD_600_ = 0.25–0.45). Total protein isolation was performed by disruption of washed cells with a French press followed by 10 cycles of 5 s of freeze–thaw (liquid nitrogen/water bath at 100 °C). To remove cell debris and unbroken cells, the lysate was centrifuged (14,000× *g*, 20 min, 4 °C). Soluble fractions (10 µg) were loaded onto 12% Mini-PROTEAN^®^ TGX™ Precast Protein Gels and run during 15 min at 100 V. Each protein sample lane was excised in 2 × 2 cm pieces. Protein samples were reduced with dithiotreitol (DTT) in 10 mM ammonium bicarbonate (45 min, 56 °C) and alkylated with iodoacetamide. Proteins were digested with trypsin. After that, the peptides were purified and desalted with ZipTip C18 columns (Millipore, Darmstadt, Germany). Peptides were analyzed by nanoLC–MS/MS in a Thermo Scientific Q-Exactive Mass Spectrometer coupled to a nanoHPLC EASY-nLC 1000 (Thermo Scientific, Waltham, MA, USA). For the LC–MS/MS analysis, approximately 1 μg of peptides was loaded onto the column and eluted for 120 min using a reverse-phase column (C18, 2 µm, 100A, 50 µm × 150 mm) Easy-Spray Column PepMap RSLC (P/N ES801) suitable for separating protein complexes with a high degree of resolution. The flow rate used for the nano column was 300 nL min^−1^ and the solvent range from 7% B (5 min) to 35% (120 min). Solvent A was 0.1% formic acid in water, whereas B was 0.1% formic acid in acetonitrile. The injection volume was 2 µL. The MS equipment has a high collision dissociation cell (HCD) for fragmentation and an Orbitrap analyzer (Thermo Scientific, Q-Exactive). A voltage of 3.5 kV was used for electrospray ionization (Thermo Scientific, EASY-SPRAY). XCalibur 3.0.63 (Thermo Scientific, Waltham, MA, USA) software was used for data acquisition. Equipment configuration allowed peptide identification at the same time as their chromatographic separation. Full-scan mass spectra were acquired in the Orbitrap analyzer. The scanned mass range was 400–1800 *m*/*z*, at a resolution of 70,000 at 400 *m*/*z*, and the 15 most intense ions in each cycle were sequentially isolated, fragmented by HCD, and measured in the Orbitrap analyzer. Peptides with a charge of +1 or with unassigned charge state were excluded from fragmentation for MS2.

Q-Exactive raw data were processed using Proteome Discoverer software (version 2.1.1.21 Thermo Scientific, Waltham, MA, USA) and searched against *Bradyrhizobium diazoefficiens* (strain JCM 10833 IAM 13628 NBRC 14792 USDA 110) UP000002526 (https://www.uniprot.org/uniprot; accessed on 17 October 2023) protein sequence database, with trypsin specificity and a maximum of one missed cleavage per peptide. Proteome Discoverer searches were performed with a precursor mass tolerance of 10 ppm and product ion tolerance to 0.05 Da. Static modifications were set to carbamidomethylation of Cys, and dynamic modifications were set to oxidation of Met and N-terminal acetylation. Protein hits were filtered for high-confidence peptide matches (at least two peptides per protein), with a maximum protein and peptide false discovery rate of 1% calculated by employing a reverse database strategy. We identified 1706 and 1693 total proteins of wild type and *phaR* mutant, respectively. The results of this analysis were imported onto Perseus (version 1.6.10.43) [69] and proteins with log_2_ FC ≥ 0.59 or ≤−0.59 (i.e., FC ≥ 1.5 or ≤1.5) and *p*-value ≤ 0.05, when comparing the *phaR* mutant and the wild type strains, were considered as differentially expressed. Proteins found in the three replicates of a strain and in none of the other were regarded as expressed and nonexpressed, respectively.

### 4.6. Overexpression and Purification of a Recombinant Untagged PhaR Protein

To obtain a recombinant nontagged PhaR derivative, the expression plasmid pMB1124 was constructed (Table S7). This plasmid encodes the PhaR protein fused to a C-terminal intein–chitin binding domain (CBD) that allows protein purification without a tag or extra amino acids according to the intein-mediated purification with an affinity chitin-binding tag (IMPACT) methodology (New England Biolabs (NEB), Hitchin, UK). For that purpose, a 623 bp PCR fragment harboring the *phaR* gene with artificially engineered *Nde*I and *Bcu*I ends was amplified with the 0227_exp_1_for/0227_exp_2_rev primers’ pair (Table S5), and subsequently cloned into pTXB1 expression vector. The correct sequence of the pMB1124 plasmid was verified by sequencing.

For protein overproduction and purification, we followed a modified protocol as the one previously described for other *B. diazoefficiens* proteins [19]. In brief, *E. coli* ER2566 cells transformed with plasmid pMB1124 were used to inoculate at an OD_600_ = 0.02, 500 mL LB cultures supplemented with ampicillin in 2 L flasks. The cultures were then incubated at 37 °C until reaching an OD_600_ = 0.3. Then, the cultures were incubated at 30 °C until an OD_600_ = 0.6–0.8 was reached. At this point, 50 µM isopropyl-β-D-thiogalactopyranoside (IPTG) was added to the cultures for protein overexpression, and incubated overnight at 16 °C. Pellets were harvested, washed once with column buffer (20 mM Na-HEPES, pH 8, 500 mM NaCl, 1 mM EDTA, 0.1% Triton X-100), and centrifuged at 5500× *g* for 7 min. Cells were then resuspended in 5 mL of column buffer supplemented with Dnase (20 µg/mL) and cOmplete Protease Inhibitor Cocktail tablets (Merck KGaA, Darmstadt, Germany), and disrupted by 4 cycles of sonication (20 MHz, 15 s) with cooling on ice. The cell extract was fractionated at 20,000× *g* for 30 min. The supernatant was then diluted 10 times before loading onto an equilibrated chitin resin column (NEB, Hitchin, UK). The column was washed with column buffer and the CBD tag cleavage was induced with 50 mM DTT overnight at room temperature. Untagged protein fractions were eluted with column buffer and the aliquots with higher protein concentration were combined before passing through a desalting column PD-10 (Cytiva Europe GmbH, Cornellá de Llobregat, Spain) for buffer exchange to mIVT buffer (40 mM Tris-HCl pH 7.0, 150 mM KCl, 0.1 mM EDTA, 0.1 mM DTT). Protein integrity and purity were checked by SDS-PAGE [70].

### 4.7. Electrophoretic Mobility Shift DNA Assays

Direct PhaR–DNA interaction was tested by EMSA assays. First, the promoter regions of selected candidates of the PhaR core regulon were amplified as ~300–400 bp fragments by PCR using suitable primers (Table S5). Double-stranded oligonucleotides harboring *phaP1*, blr2131, and blr4358 promoter derivatives were generated by mixing equal volumes of 100 μM solutions of complementary single-stranded oligonucleotides, heating at 100 °C for 30 min, and slowly cooling to 25 °C. The correct annealing of the double-stranded oligonucleotides was validated according to the method described by Goedhart and Gadella [71]. Briefly, samples were loaded onto a 3% agarose gel in 10 mM sodium borate buffer and subsequently electrophoresed at 300 V for 10 min.

EMSA assays were performed in 10 µL reactions containing either 2 ng of purified PCR fragments spanning selected promoter regions or of double-stranded oligonucleotides (Table S5), and different protein concentrations ranging from 0 to 2 µM in mIVT buffer. The reactions were incubated for 30 min at room temperature and subsequently mixed with one-sixth volume of loading dye (30% glycerol in mIVT buffer supplemented with bromophenol blue) prior loading onto a 6% nondenaturing polyacrylamide-0.5X Tris-Borate EDTA (TBE) gel. After electrophoresis at 180 V for 30 min, the gels were incubated in a 0.01% SYBR-Gold (Invitrogen, Waltham, MA, USA) solution in 0.5X TBE for 15 min. Finally, UV-induced signals were detected by a Gel Doc XR+ System (Bio-Rad, Hercules, CA, USA) and quantified using the Quantity One and Image Lab 6.1 software (Bio-Rad, Hercules, CA, USA).

### 4.8. Gel Filtration

The oligomeric state of purified recombinant untagged PhaR protein was determined by analytical SEC experiments on a Superdex 75 10/300 GL column (GE Healthcare, Uppsala, Sweden). In these experiments, the pH of the mIVT buffer (protein and column) was increased to 8, as this pH improved the solubility of the PhaR protein in solution. After equilibration of the column with buffer, protein sample aliquots of 200 µL at a concentration ranging 5 to 30 µM were injected and separated at a flow rate of 0.5 mL min^−1^ on an ÄKTA^TM^ purifier Fast Protein Liquid Chromatography (FPLC) purification system (Pharmacia Biotech, Uppsala, Sweden). The absorption profile of the eluent was simultaneously recorded at 220 and at 280 nm. The following proteins were used as standard for calibration (Figure S4): conalbumin (75 kDa), carbonic anhydrase (29 kDa), ribonuclease A (13.7 kDa), and aprotinin (6.5 kDa) (Cytiva, Little Chalfont, UK). Gel filtration experiments were repeated at least twice with independent preparations over a range of at least three concentrations. The UNICORN™ system control software version 5.11 (GE Healthcare, Uppsala, Sweden) was employed to program the chromatography runs and for preliminary analyses.

### 4.9. PHB Granules Isolation and Associated Protein Analysis

PHB granules and their associated proteins were isolated according to methodology by Pötter and coworkers [72] with some modification. First, 400 mL (10 cultures × 40 mL) of *B. diazoefficiens* wild type grown microoxically in Götz mineral medium with mannitol as the sole carbon source were prepared for each PHB granule extraction. Briefly, the pellet obtained after centrifugation (10 min at 7000 rpm) was resuspended in 20 mL of potassium phosphate buffer (BP, 100 mM, pH 7.5) and lysed by French press (three passages of 100 × 10^6^ Pa). Then, the lysate was centrifuged (4000× *g* 10 min) and resuspended in 10 mL BP buffer. The sample was placed on the first gradient (8 mL of 50% glycerol and 2 mL of 90% glycerol) and centrifuged at 210,000× *g* (Sorvall WX Ultra 100, fixed angle rotor T1250, Thermo Scientific, Waltham, MA, USA) at 4 °C for 2 h. The remaining sample above the 90% glycerol was transferred to a new tube and 50 mL of BP buffer were added for washing. Subsequently, the sample was centrifuged (30,000× *g* for 10 min) and the precipitate was resuspended in 5 mL of BP buffer. The total volume of 5 mL was deposited on the second gradient (2 mL of 50% glycerol, 2 mL of 60% glycerol, 2 mL of 80% glycerol and 2 mL of 90% glycerol) and centrifuged at 210,000× *g* (Sorvall WX Ultra 100, fixed angle rotor T1250, Thermo Scientific, Waltham, MA, USA) at 4 °C for 2 h. The material on the 90% glycerol was removed and washed three times with 150 mM NaCl and three times with mQ water, followed by centrifugation (14,000× *g* for 20 min) and resuspension in 200 μL of 50% glycerol, and stored at −20 °C. PHB content was measured by spectrophotometry, using commercial PHB (Sigma-Aldrich, Saint, Louis, MO, USA) as standard [2]. An additional treatment after the last ultracentrifugation step, i.e., 3 washes with Triton-X 100 in 100 mM potassium phosphate buffer, pH 7.5, and 3 washes with ultrapure water, did not help to exclude possible false-positive PHB granule-binding proteins in our assays, since the total number of proteins isolated from PHB granules was similar.

PHB granules associated proteins were separated from PHB by boiling in 1X Laemmli buffer (4% SDS, 20% glycerol, 10% 2-mercaptoethanol, 0.004% bromophenol blue and 0.125 M Tris HCl) for 5 min. After centrifugation, the supernatant was loaded onto an SDS-PAGE gel, and run for 15 min. The gel was stained with R-250 Coomassie Blue. Gel bands with proteins were excised in two spots, reduced with DTT, and treated separately with iodoacetamide and trypsin digested during 18 h at 30 °C. Peptides were extracted with 0.2% TFA, 30% AcN. Eluted peptides were dried in a Speed-Vac and stored at −20 °C. Samples were analyzed by nLC (easy nanoLC; Proxeon, Odense, Denmark) coupled with spectrometer ion trap masses (Amazon Speed ETD; Bruker, Bremen, Germany). Protein identification was performed using the Protein Scape program (Bruker, Bremen, Germany) and MASCOT (Matrix Science, London, UK). Searches were performed against *B. diazoefficiens* (strain JCM 10833 IAM 13628 NBRC 14792 USDA 110) UP000002526 (https://www.uniprot.org/uniprotkb?query=UP000002526; accessed on 13 November 2023) protein sequence database, considering carbamidomethylation as a fixed modification, and oxidation as a variable modification.

### 4.10. Databases

The *B. diazoefficiens* USDA 110 genome (T00109) was visualized at https://www.genome.jp/entry/gn:T00109; accessed on 2 October 2023. Gene functions were predicted using the Kyoto Encyclopedia of Genes and Genomes [73].

Protein functions were predicted using the Universal Protein Knowledgebase in 2023 [74]. The *B. diazoefficiens* USDA 110 (UP000002526) proteome was downloaded from https://www.uniprot.org/uniprotkb?query=UP000002526; accessed on 13 November 2023).

## Figures and Tables

**Table 1:** List of the 30 gene-associated promoters employed for PhaR-binding sites prediction. Note that their expression is, at the same time, upregulated at transcriptional and at protein levels, in the *phaR* mutant in comparison with the wild type (WT), both grown in Götz minimal medium supplemented with mannitol under microaerobic conditions (0.5% O_2_).

Locus tag ^a^	Gene Name ^b^	Protein Code ^c^	Description ^d^	FC (*phaR* vs. WT) ^e^	log_2_ FC (*phaR* vs. WT) ^f^	Position ^g^	Motif ^h^	Predicted Operon Structure ^i^
blr0515	*sdhB*	H7C6L4	succinate dehydrogenase iron-sulfur protein subunit	2.6	1.4	−79	GGCGATGCATCG	-
blr0697	*-*	Q89WJ0	hypothetical protein	2.3	2.3	−269 −243 −206 −172	TGCCGCGCGACT GGCGTTGCGAAC GGCTGAGCGCGG GGCCGCGCGCGA	blr0697–blr0698
bll0957	*-*	Q89VT8	hypothetical protein	3.0	2.5	−235−228	TGCGATGCGCAC CGCACCGCGGGC	*-*
bll0958	*acd*	Q89VT7	acyl-CoA dehydrogenase	3.0	1.7	−52	GGCATCGCGAAT	*-*
bll1105	*metZ*	Q89VE2	O-succinylhomoserine sulfhydrylase	2.3	1.5	-	-	*-*
blr1496	*-*	Q89UC1	unknown protein	2.1	19.9	−156 −48	GGCGCGGCGCTG TGCGCTGCGCCT	*-*
blr2221	*bioA*	Q89T29	adenosylmethionine-8-amino-7-oxononanoate aminotransferase	2.3	1.1	−189 −176	CGCTTGGCAAGC CGCGCTGCGACA	*-*
blr3456	*-*	Q89PM4	hypothetical protein	7.2	7.2	−97 −92	AGCGCGGCGTCG GGCGTCGCAGGC	blr3456–blr3459
bll3830	*-*	Q89NK8	hypothetical protein	2.3	1.0	−114 −30	AGCTATGCGTCG AGCGTTGCAGTT	*-*
blr3954	*-*	Q89N88	methylmalonate-semialdehyde dehydrogenase	3.7	4.4	−165	CGCCCCGCACGA	blr3954–blr3957
blr3958	*-*	Q89N84	putative acetyl-coenzyme A synthetase (EC 6.2.1.1)	2.6	2.4	−255 −199 −194	GGCGCGGCGACG GGCGGGGCAGGG GGCAGGGCGGGG	*-*
bll4252	*-*	Q89ME1	putative hydrolase	3.7	2.9	−188	AGCGAAGCGGCC	*-*
blr4257	*-*	Q89MD6	putative hydrolase	7.0	3.1	−57 −52	CGCTCCGCATCG CGCATCGCACCC	blr4257–blr4264
blr4358	*-*	Q89M35	similar to N-acetyl-gamma-glutamyl-phosphate reductase	8.7	2.6	−119	CCGCCAAGATCAAGGCCGCGAA	*-*
blr4582	***argD2***	Q89LG2	acetylornithine aminotransferase	48.9	18.2	−286 −259 −243 −170	AGCTGCGCATCC CGCTCGGCGGGC CGCGAAGCGCCC CGCCGCGCAGCT	*-*
blr4680	*-*	Q89L68	hypothetical protein	2.8	1.6	−235 −228 −32	CGCCATGCGCTA CGCTACGCGGAC GGCCTCGCGCGA	*-*
blr4687	*asd*	Q89L61	aspartate-semialdehyde dehydrogenase	9.1	22.3	−209	CCGCCATGTTCACGACCACCAA	*-*
bll4788	*-*	Q89KW2	hypothetical protein	2.0	2.4	−186 −68	GGCCGTGCACCG AGCGAAGCGGGG	*-*
bll4896	*-*	Q89KK9	ABC transporter substrate-binding protein	2.4	1.1	−107	AGCGTTGCAAGG	*-*
bll5081	*-*	Q89K37	putative multidrug resistance protein	7.1	2.0	-	-	bll5081–bll5079
bll5155	***phaP1***	Q89JW4	**phasin family protein**	2.4	0.9	−101 −72	TGCGACGCACAA TGCGTTGCACTA	*-*
bll5290	*-*	Q89JJ3	hypothetical protein	2.3	2.0	−298 −123 −41	GGCGAGGCATCC AGCAGCGCGCGA CGCCAAGCGGCC	*-*
bll5331		Q89JF2	hypothetical protein	2.1	0.7	−267 −252 −119 −42 −40	TGCCGCGCGACT GGCGTTGCGAAC GGCGCGGCGGTT CGCGCTGCGCGC CGCTGCGCGCGC	bll5331–bll5330
bll5846	*-*	Q89HZ3	hypothetical protein	2.8	1.8	−177 −174	GGCGGCGCGGCG GGCGCGGCGACG	*-*
bll5913	*cycC*	Q45234	cytochrome C	3.2	0.8	−114 −97	TGCCGCGCGGGA CGCCCCGCGCGG	*-*
blr6331	*bkdA1*	Q89GL4	2-oxoisovalerate dehydrogenase alpha subunit	9.9	8.1	−234 −46	CGCGGCGCGGTT GGCCAGGCGTTG	blr6331–blr6334
blr6667	*-*	Q89FN2	hypothetical protein	2.7	1.2	−28	GGCCCTGCACGA	*-*
bsr6998	*-*	Q89ES4	hypothetical protein	2.5	2.8	−253 −246 −216 −208 −75 −55	GGCCTCGCGCTC CGCTCCGCGCGC AGCTTTGCAGCG AGCGTCGCGCTC GGCTTAGCGGAT CGCCGCGCGTAG	*-*
blr7054	*-*	Q89EL9	hypothetical protein	6.6	0.7	−194 −25	GGCATCGCGCTG TGCTGCGCGCTC	*-*
bll7459	*-*	Q89DI0	probable branched-chain amino acid aminotransferase protein	2.2	1.0	-	-	*-*

^a^ Nomenclature of the *B. diazoefficiens* USDA 110 genes according to [4] (GenBank acc. # NC_004463.1; RefSeq annotation as of November 2016). Confirmed direct PhaR targets validated by electrophoretic mobility shift DNA assays (EMSA) are shaded in grey. ^b^ Gene name according to the NCBI annotation with modifications (boldfaced) (GenBank acc. # NC_004463.1; RefSeq annotation; accessed on 23 November 2016). ^c^ Protein/gene product accession number according to the UniProt database (https://www.uniprot.org/; accessed on 17 October 2023). ^d^ Protein/gene product according to the NCBI annotation with modifications (boldfaced) (GenBank acc. # NC_004463.1; RefSeq annotation; accessed on 23 November 2016). ^e^ Fold change (FC) values of gene expression in the *phaR* mutant strain compared to the WT both cultured microoxically in transcriptomic experiments. Only differentially expressed genes with FC ≥ 2 were considered. ^f^ Log_2_ fold change (FC) values from the comparison of *phaR* mutant cells with wild-type cells, both grown microoxically in proteomic experiments. Only differentially expressed proteins with log_2_ FC ≥ 0.59, i.e., FC ≥ 1.5, were considered. ^g^ Position of the first nucleotide of the motif relative to the annotated translational start site of the associated gene. ^h^ Predicted PhaR-binding sites. ^i^ Operon structure prediction according to [17].

## Data Availability

Microarray data are available upon acceptance of this article via the Gene Expression Omnibus (GEO) series record GSE250298 at the National Center for Biotechnology Information (NCBI) GEO platform ([75]; http://www.ncbi.nlm.nih.gov/geo; accessed on 15 December 2023). The mass spectrometry proteomics data have been deposited to the ProteomeXchange Consortium via the PRIDE [76] partner repository with the dataset identifier PXD047760 for LFQ proteomics (accessed on 12 December 2023) and PXD047962 for nano-LC–MS/MS (accessed on 19 December 2023). Other additional data are provided in the Supplementary Material.

## Supplementary Materials

The following supporting information can be downloaded at: https://www.mdpi.com/1422-0067/25/4/2157/s1.
