# Supplementary material for: Pleiotropic Effects of PhaR Regulator in Bradyrhizobium diazoefficiens Microaerobic Metabolism
Source: Int J Mol Sci. 2024 Feb 10;25(4):2157. doi: 10.3390/ijms25042157 (PMC10888616; doi:10.3390/ijms25042157)
Supplement: Supplementary file 1 [file ijms-25-02157-s001.zip › Quelas_et_al_Legends_to_Supplementary_Figures_and_Tables.pdf]

Quelas *et al.*, Legends to Supplementary Figures and Tables

Figure S1. SDS-PAGE analysis of PhaR recombinant purified protein. Samples of representative steps during protein overexpression and purification were monitored in Coomassie blue-stained 14% SDS-PAGE gels. Extracts of uninduced (lane 1) and induced (lane 2) *E. coli* ER2566 overexpressing cells. French press disrupted cells were centrifugated to obtain the soluble fraction (lane 3) which was loaded onto a chitin resin column, and the PhaR-*Mxe* GyrA-Intein-CBD recombinant protein was immobilized and removed from the flow through (lane 4). Non-tagged PhaR protein was eluted after DTT-induced cleavage overnight at room temperature (lanes 5, 6, and 7). The calculated M_w of the non-tagged (22.6 kDa) and C-terminally tagged PhaR protein (50.8 kDa) are shown on the right margin. The size of bands of the molecular marker PageRuler™ Prestained Protein Ladder (ThermoFisher Scientific, Waltham, MA, USA) (M) are shown on the left margin.

Figure S2. Interaction of PhaR protein with *fixR* and *fixK*$_2$ promoters tested by EMSAs. Assays were performed with increasing PhaR protein concentration (0, 0.5, 1 and 2 μM) and 2 ng of a PCR product comprising the corresponding promoter region (*fixR* or *fixK*$_2$). The black arrow indicates the position of PhaR-DNA complexes, the white arrow indicates the position of free DNA. The molecular marker GeneRuler 1 kb Plus DNA Ladder (ThermoFisher Scientific, Waltham, MA, USA) (M) is shown on the left margin. bp, base pairs.

Figure S3. Functional mutagenesis of blr2131 and blr4358 promoters. PhaR interaction with different promoter variants was analyzed by EMSA experiments. Transversion of G residues to T residues and C residues to A residues were introduced at positions 2, 3, 7, and 8 of each of the three predicted 12-pb boxes present at the blr2131 promoter (panels A-D), and at invariable positions of the 22-bp pattern at the blr4538 promoter (positions 1, 2, 3, 4, 5, 8, 11, 14, 16, 17, 19; panels E-F). Double-stranded oligonucleotides were incubated with a series of purified PhaR protein concentrations (0, 0.5, 1, and 2 μM) indicated at the top of each group of gels. Protein-DNA interaction was analyzed in SYBR-Gold-stained agarose gel electrophoresis. Black arrows, PhaR-DNA complexes; white arrows, free DNA.

Figure S4. Size exclusion chromatography (SEC) calibration curve. (A) The following standards were used: conalbumin (CO, 75 kDa), carbonic anhydrase (CA, 29 kDa), ribonuclease A (R, 13.7 kDa), aprotinin (AP, 6.5 kDa). The void volume of the column was determined as 7.89 mL using blue dextran 2000 (BD, 2,000 kDa). The end of the run end was indicated by the elution volume of acetone (AC). Chromatograms were obtained at 0.5 mL/min using a Superdex 75 10/300 GL column (GE Healthcare, Uppsala, Sweden). (B) The regression formula for M_w calculation was obtained by plotting K_{av} vs. M_w (Log_{10} scale) of each standard. K_{av}, ratio of the elution volume of each standard (V_e) to the total volume of the column (24 mL).

Table S1. Compilation of microarray data analyses performed in this study. (Datasheet A) 733 genes whose expression is decreased in the *phaR* mutant compared to the wild type, both grown in Götz medium supplemented with mannitol under microaerobic conditions (0.5% O_2), fold change (FC) ≤ −2. (Datasheet B) 482 genes whose expression is increased in the *phaR* mutant compared to the wild type, both grown in Götz medium supplemented with mannitol under microaerobic conditions (0.5% O_2), FC ≥ 2. The "Overview" sheet provides explanations to the individual gene groups listed in Datasheets A-B as well as the associated references.

Table S2. Compilation of proteomics Label-Free Quantification (LFQ) performed in this study. (Datasheet A) 232 proteins whose abundance is decreased in the *phaR* mutant in comparison with the wild type, both grown in Götz medium supplemented with mannitol under microaerobic conditions (0.5% O_2), log_2 fold change (FC) ≤ −0.59. (Datasheet B) 294 proteins whose abundance is increased in the *phaR* mutant in comparison with the wild type, both grown in Götz medium supplemented with mannitol under microaerobic conditions (0.5% O_2), log_2 FC ≥ 0.59. The "Overview" sheet provides explanations to the individual protein groups listed in Datasheets A-B as well as the associated references.

Table S3. List of genes/proteins associated to carbon and nitrogen metabolism whose expression is regulated at transcriptional and/or at protein levels in the *phaR* mutant compared to the wild type, both grown in Götz minimal medium supplemented with mannitol under microaerobic conditions (0.5% O_2).

Table S4. Survey of the 78 promoters with downregulated expression, at both at transcriptional and at protein levels, in the *phaR* mutant in comparison with the wild type (WT), both grown in Götz minimal medium supplemented with mannitol under microaerobic conditions (0.5% O_2).

Table S5. Oligonucleotides employed in this work.

Table S6. PHB granule-associated proteins detected in wild type strain grown in Götz medium supplemented with mannitol under microaerobic conditions (0.5% O_2).

Table S7. Strains and plasmids used in this study.
