# Supplementary material for: Pleiotropic Effects of PhaR Regulator in Bradyrhizobium diazoefficiens Microaerobic Metabolism
Source: Int J Mol Sci. 2024 Feb 10;25(4):2157. doi: 10.3390/ijms25042157 (PMC10888616; doi:10.3390/ijms25042157)
Supplement: Supplementary file 1 [file ijms-25-02157-s001.zip › Quelas_et_al_Table S5.docx]

**Table S5.** Oligonucleotides employed in this work.

| **Primer name** | **DNA sequence (5’ → 3’)** ^(a,b,c,d)^ | **Source or**  **reference** |
| --- | --- | --- |
| **PhaR purification** | | |
| 0227_exp_1_for | ACGCATATGGCGAAATCAGACCAACC | This work |
| 0227_exp_1_rev | TGCAAGCTTCCTACTCTTCCTTCTTCGAC | This work |
| 0227_exp_2_rev | GAT[ACTAGTGCATCTCCCGTGATGCA]_INTEIN_CTCTTCCTTCTTCGACATCC | This work |
| **RNA quality check prior to cDNA synthesis** | | |
| fixN4_for | CGGGATCCCGACTCCTATCCGGTCGAGGAC | [1] |
| fixN4_rev | CGGAATTCCGGGTCATGGATTTGGAGATGG |  |
| \| **EMSA experiments** \| \| \| \| --- \| --- \| --- \| \| 1755_EMSA_for \| gtcgcgcctttgaatacg \| This work \| \| 1755_EMSA_rev \| AAGATGAAATCGCGCTCTTG \| \| 2036_EMSA_for \| GTTGTGCGATCGTGAAAG \| This work \| \| 2036_EMSA_rev \| CATTTGGCAGATCGAGAC \| \| 2037_EMSA_for \| GTGGCCAGCATCTGTGAAG \| This work \| \| 2037_EMSA_rev \| TGGACCCATGTCCGGATTG \| \| 2131_EMSA_for \| ggctacggcatcaagagttt \| This work \| \| 2131_EMSA_rev \| GGCAGCTCTTGTCGGATTTA \| \| 2757_EMSA_for \| CGGGATCCCGGTAAATCGGACGGCGAAAC \| This work \| \| 2757_EMSA_rev \| TGCTCTAGATTGCCACCGATTTGGGTGTTG \| \| 2887_EMSA_for \| gatgtcgacggcaagacc \| This work \| \| 2887_EMSA_rev \| CAGTGGTCATggggttctct \| \| 3010_EMSA_for \| cgtcggcctgttctgttt \| This work \| \| 3010_EMSA_rev \| ATGTCTGGATTTGGGCTGTG \| \| 3464_EMSA_for \| aaggcatcgttcgcattg \| This work \| \| 3464_EMSA_rev \| GCCATgacgttactccctgt \| \| 3530_EMSA_for \| ctgcggtcgaacagtttttg \| This work \| \| 3530_EMSA_rev \| GGCTGACGTCCTGCATatct \| \| 3871_EMSA_for \| cggtctaccgtccgatcac \| This work \| \| 3871_EMSA_rev \| TCGGACGATCGATGAAGAAT \| \| 3872_EMSA_for \| gtagaggctgggcggatt \| This work \| \| 3872_EMSA_rev \| CAGGGCGAGAGGTATTTTGG \| \| 4358_EMSA_for \| gctgatggaggaggtcgat \| This work \| \| 4358_EMSA_rev \| GTTCACcgtgacgggatagt \| \| 4582_EMSA_for \| ctcatcgacagcgcgatt \| This work \| \| 4582_EMSA_rev \| GTGATATCCATCAGCGCATC \| \| 4655_EMSA_for \| tgatgttctcggacatggaa \| This work \| \| 4655_EMSA_rev \| CGATTTCGAGGAAGGATCTG \| \| 4687_EMSA_for \| gcccggacgaagtagagac \| This work \| \| 4687_EMSA_rev \| ACGACGGGATCGTTACTCAC \| \| 5155_1_EMSA_for \| GAACCTGAGCCGCTATTCTG \| This work \| \| 5155_1_EMSA_rev \| CGAACTGCTCTTTCCCGTAG \| \| 6073_EMSA_for \| gtcgtcgaccaccgtgag \| This work \| \| 6073_EMSA_rev \| CTCATggctgaatgatcctg \| \| 6331_EMSA_for \| aacgtgacatgcaacctcaa \| This work \| \| 6331_EMSA_rev \| GAACGTGAAAGCGAAGTGG \| \| 7395_EMSA_for \| gtagcccgatggattctcag \| This work \| \| 7395_EMSA_rev \| tcacgatcatggaaaatgga \| | | |
| **EMSA experiments with mutated PhaR box** | | |
| blr2131_EMSA_1_for | TGGTCTCAAAAATGCGAAGCATCTCTTGCGGGGCGGCCGCCGGCGTCTCGCCTTTTTGGAGCAACGCAGGTGCATACTGAAGG | This work |
| blr2131_EMSA_1_rev | CCTTCAGTATGCACCTGCGTTGCTCCAAAAAGGCGAGACGCCGGCGGCCGCCCCGCAAGAGATGCTTCGCATTTTTGAGACCA |  |
| blr2131_EMSA_2_for | TGGTCTCAAAAAT**TAT**AA**TA**AT**A**TCTTGCGGGGCGGCCGCCGGCGTCTCGCCTTTTTGGAGCAACGCAGGTGCATACTGAAGG | This work |
| blr2131_EMSA_2_rev | CCTTCAGTATGCACCTGCGTTGCTCCAAAAAGGCGAGACGCCGGCGGCCGCCCCGCAAGA**T**AT**TA**TT**ATA**ATTTTTGAGACCA |  |
| blr2131_EMSA_3_for | TGGTCTCAAAAATGCGAAGCATCTCTT**TATTTTATTAA**GCCGGCGTCTCGCCTTTTTGGAGCAACGCAGGTGCATACTGAAGG | This work |
| blr2131_EMSA_3_rev | CCTTCAGTATGCACCTGCGTTGCTCCAAAAAGGCGAGACGCCGGC**TTAATAAAATA**AAGAGATGCTTCGCATTTTTGAGACCA |  |
| blr2131_EMSA_4_for | TGGTCTCAAAAATGCGAAGCATCTCTTGCGGGGCGGCCGCCGGCGTCTCGCCTTTTTGGA**TA**AA**ATA**A**TT**TGCATACTGAAGG | This work |
| blr2131_EMSA_4_rev | CCTTCAGTATGCA**AA**T**TAT**TT**TA**TCCAAAAAGGCGAGACGCCGGCGGCCGCCCCGCAAGAGATGCTTCGCATTTTTGAGACCA |  |
| blr4358_EMSA_1_for | CGCCGGATCAGCCCGCCAAGATCAAGGCCGCGAAGAAGGTCTCCAA | This work |
| blr4358_EMSA_1_rev | TTGGAGACCTTCTTCGCGGCCTTGATCTTGGCGGGCTGATCCGGCG |  |
| blr4358_EMSA_2_for | CGCCGGATCAGC**AATAA**AA**T**AT**A**AA**TTAATAT**AAGAAGGTCTCCAA | This work |
| blr4358_EMSA_2_rev | TTGGAGACCTTCTT**ATATTAA**TT**T**AT**A**TT**TTATT**GCTGATCCGGCG |  |
| 5155_2_EMSA_for | GATCTAGTTTTTGT**GC**GAC**GC**ACAAGATTCTTGACTTTTTTGT**GC**GTT**GC**ACTAATTGTGGGCAGAG | This work |
| 5155_2_EMSA_rev | CTCTGCCCACAATTAGT**GC**AAC**GC**ACAAAAAAGTCAAGAATCTTGT**GC**GTC**GC**ACAAAAACTAGATC |  |
| 5155_3_EMSA_for | GATCTAGTTTTTGT**TA**GAC**TA**ACAAGATTCTTGACTTTTTTGT**GC**GTT**GC**ACTAATTGTGGGCAGAG | This work |
| 5155_3_EMSA_rev | CTCTGCCCACAATTAGT**GC**AAC**GC**ACAAAAAAGTCAAGAATCTTGT**TA**GTC**TA**ACAAAAACTAGATC |  |
| 5155_4_EMSA_for | GATCTAGTTTTTGT**GC**GAC**GC**ACAAGATTCTTGACTTTTTTGT**TA**GTT**TA**ACTAATTGTGGGCAGAG | This work |
| 5155_4_EMSA_rev | CTCTGCCCACAATTAGT**TA**AAC**TG**ACAAAAAAGTCAAGAATCTTGT**GC**GTC**GC**ACAAAAACTAGATC |  |
| 5155_5_EMSA_for | GATCTAGTTTTTGT**TA**GAC**TA**ACAAGATTCTTGACTTTTTTGT**TA**GTT**TA**ACTAATTGTGGGCAGAG | This work |
| 5155_5_EMSA_rev | CTCTGCCCACAATTAGT**TA**AAC**TG**ACAAAAAAGTCAAGAATCTTGT**TA**GTC**TA**ACAAAAACTAGATC |  |
| 5155_2_EMSA_for | GATCTAGTTTTTGT**GC**GAC**GC**ACAAGATTCTTGACTTTTTTGT**GC**GTT**GC**ACTAATTGTGGGCAGAG | This work |
| 5155_2_EMSA_rev | CTCTGCCCACAATTAGT**GC**AAC**GC**ACAAAAAAGTCAAGAATCTTGT**GC**GTC**GC**ACAAAAACTAGATC |  |

^a^ Artificial restriction sites are underlined.

^b^ Mutations are shown in boldface letters.

^c^ Additional sequences are indicated in the brackets.

^d^ PhaR boxes are show in light (short) and dark (long) grey.

**References**

1. Parejo, S.; Cabrera, J.J.; Jimenez-Leiva, A.; Tomas-Gallardo, L.; Bedmar, E.J.; Gates, A.J.; Mesa, S. Fine-tuning modulation of oxidation-mediated posttranslational control of *Bradyrhizobium diazoefficiens* FixK_2_ transcription factor. *Int. J. Mol. Sci.* **2022**, *23*, 5117.
