# Supplementary material for: Pleiotropic Effects of PhaR Regulator in Bradyrhizobium diazoefficiens Microaerobic Metabolism
Source: Int J Mol Sci. 2024 Feb 10;25(4):2157. doi: 10.3390/ijms25042157 (PMC10888616; doi:10.3390/ijms25042157)
Supplement: Supplementary file 1 [file ijms-25-02157-s001.zip › Quelas_et_al_Table S7.pdf]

Table S7. Strains and plasmids used in this study.

Strains and plasmids	Description	Resistance	Source or reference
**Strains**			
Escherichia coli			
DH5*α*	*supE*44 Δ*lacU*169 (φ80 *lacZ* ΔM15) *hsdR*17 *recA1 endA1 gyrA*96 *thi*-1 *relA*1		Bethesda Research Laboratories Inc., Gaithersburg, MD, USA
ER2566	*fhuA2 lacZ::T7 gene1 [lon] ompT gal sulA11 R(mcr-73::miniTn10*-Tets*)2 [dcm] R(zgb-210::Tn10*-Tet^S) *endA1* Δ*(mcrC-mrr)114::IS10*		New England Biolabs, USA
Bradyrhizobium diazoefficiens			
USDA 110	*B. diazoefficiens* wild-type	Cm^r	USDA culture collection
LP 3004	*B. diazoefficiens* USDA 110 spontaneous Sm^R	Cm^r Sm^r	[1]
USDA 110*spc*4	*B. diazoefficiens* USDA 110 spontaneous Sp^R	Cm^r Sp^r	[2]
LP 0227 (*phaR* mutant)	*B. diazoefficiens* LP 3004 with pIQ36 inserted in blr0227 (*phaR*) gene	Cm^r Sm^r	[1]
LP 6073 (Δ*phaC2*)	*B. diazoefficiens* USDA 110 derivative with bll6073 (*phaC2*) gene replaced by *nptI* cassette	Cm^r Km^r	[3]
**Plasmids**			
pET28a(+)	Expression vector for his-tagged protein purification, under T7 promoter control	Km^r	Novogen Inc.
pTXB1	Expression vector for the IMPACT protein purification system. It codes for a C-terminal thiol-cleavable *Mxe* GyrA-Intein-chitin-binding domain (CBD) under T7 promoter control	Ap^r	New England Biolabs, USA
pMB1124	(pTXB1) a 623 fragment NdeI-BcuI with *phaR* entire gene fused *in frame* with intein-CBD tag for native PhaR purification	Ap^r	This work

References

1. Quelas J.I.; Mesa, S.; Mongiardini, E.J.; Jendrossek, D.; Lodeiro A.R. Regulation of polyhydroxybutyrate synthesis in the soil bacterium *Bradyrhizobium diazoefficiens*. *Appl Environ Microbiol* **2016,** 30, *82*, 4299-4308.
2. Regensburger, B.; Hennecke, H. RNA polymerase from *Rhizobium japonicum*. *Arch Microbiol* **1983**, *135*, 103-119.
3. Quelas J.I.; Mongiardini, E.J.; Pérez Giménez, J.; Parisi, G.; Lodeiro A.R. Analysis of two polyhydroxyalkanoate synthases in *Bradyrhizobium japonicum* USDA 110. *J Bacteriol* **2013,** *195*, 3145-3155.
