# Supplementary figures and images for: Pleiotropic Effects of PhaR Regulator in Bradyrhizobium diazoefficiens Microaerobic Metabolism
Source: Int J Mol Sci. 2024 Feb 10;25(4):2157. doi: 10.3390/ijms25042157 (PMC10888616; doi:10.3390/ijms25042157)

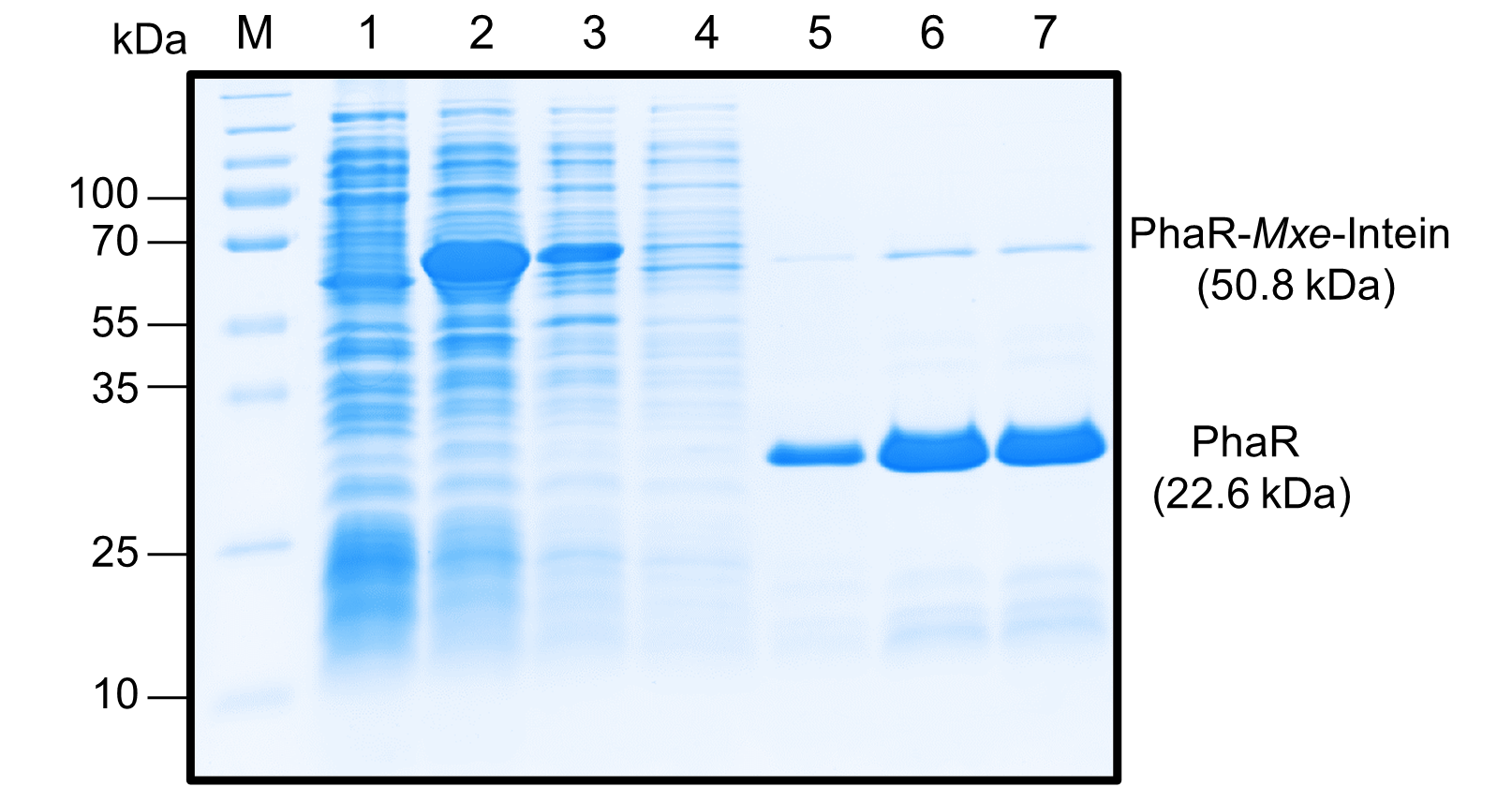

Supplement: Supplementary file 1 [file ijms-25-02157-s001.zip › Quelas_et_al_Figure S1.tif]

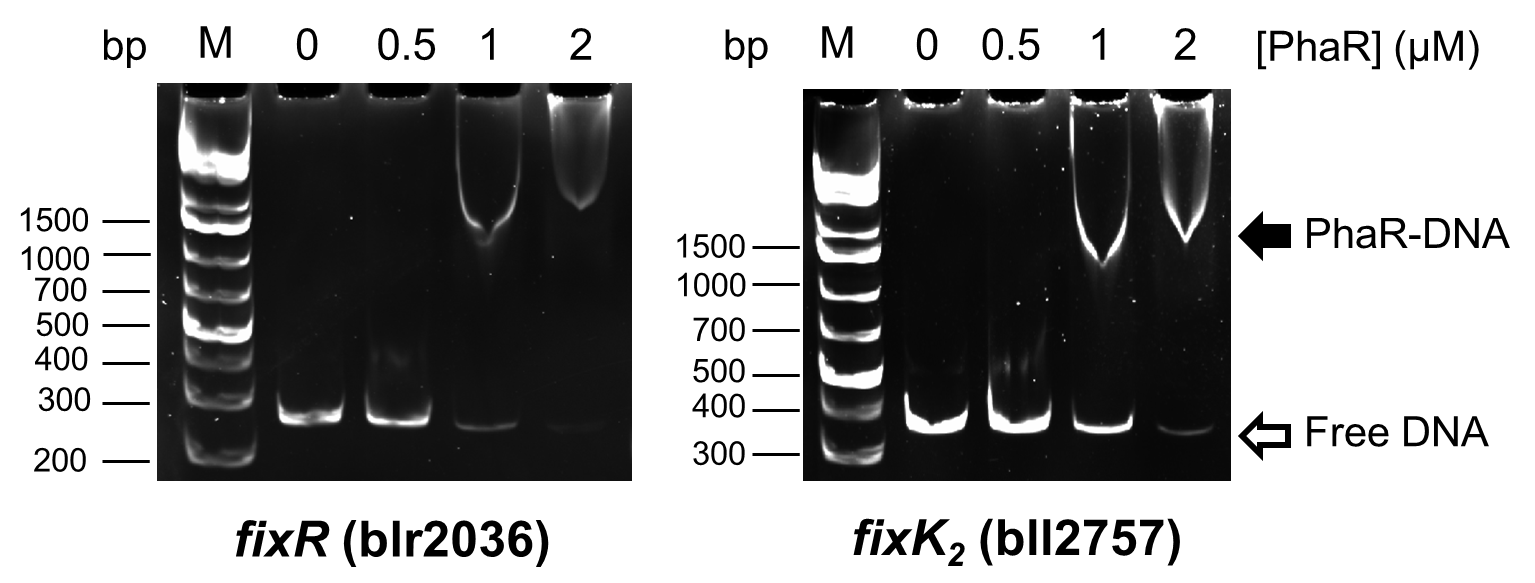

Supplement: Supplementary file 1 [file ijms-25-02157-s001.zip › Quelas_et_al_Figure S2.tif]

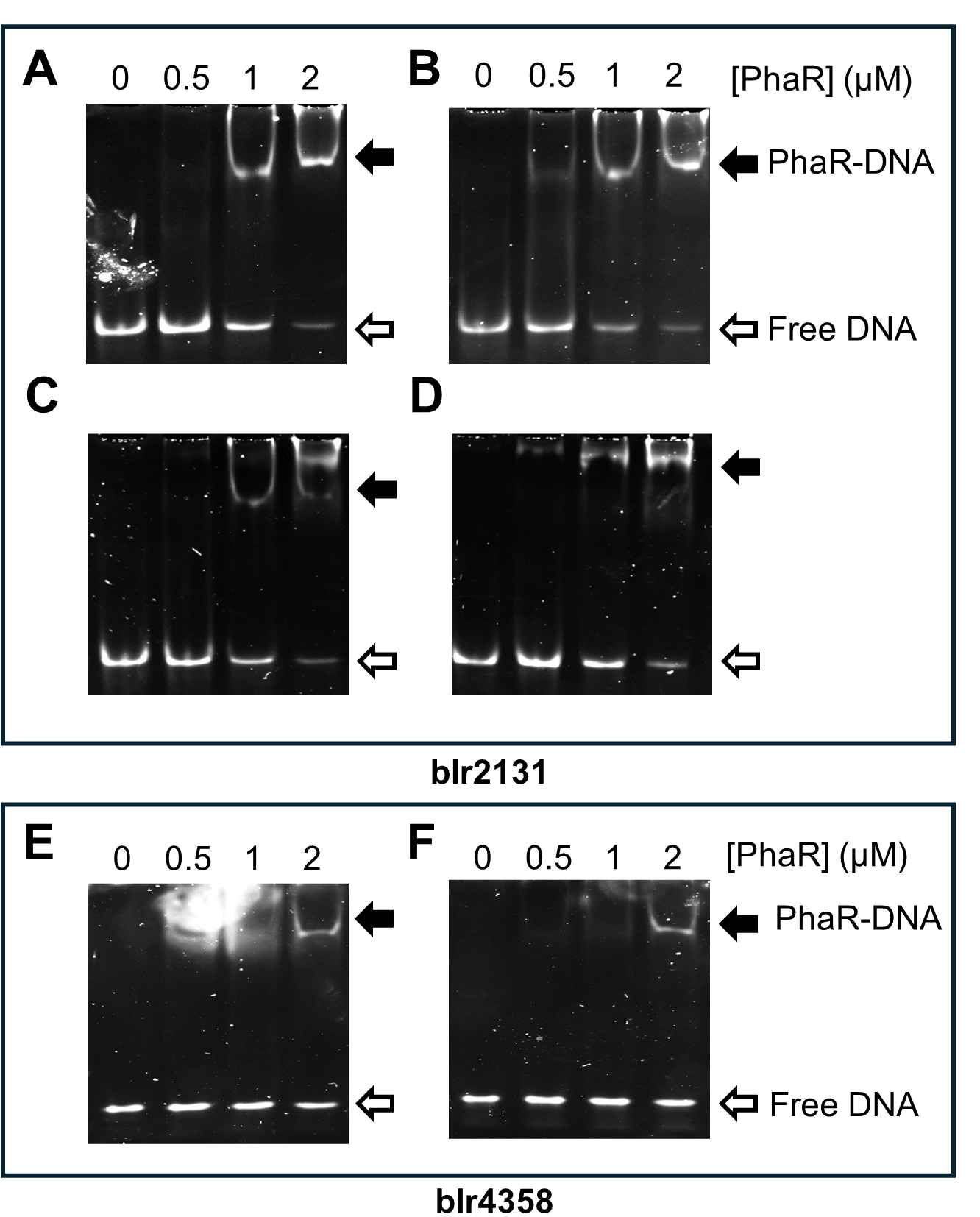

Supplement: Supplementary file 1 [file ijms-25-02157-s001.zip › Quelas_et_al_Figure S3.tif]

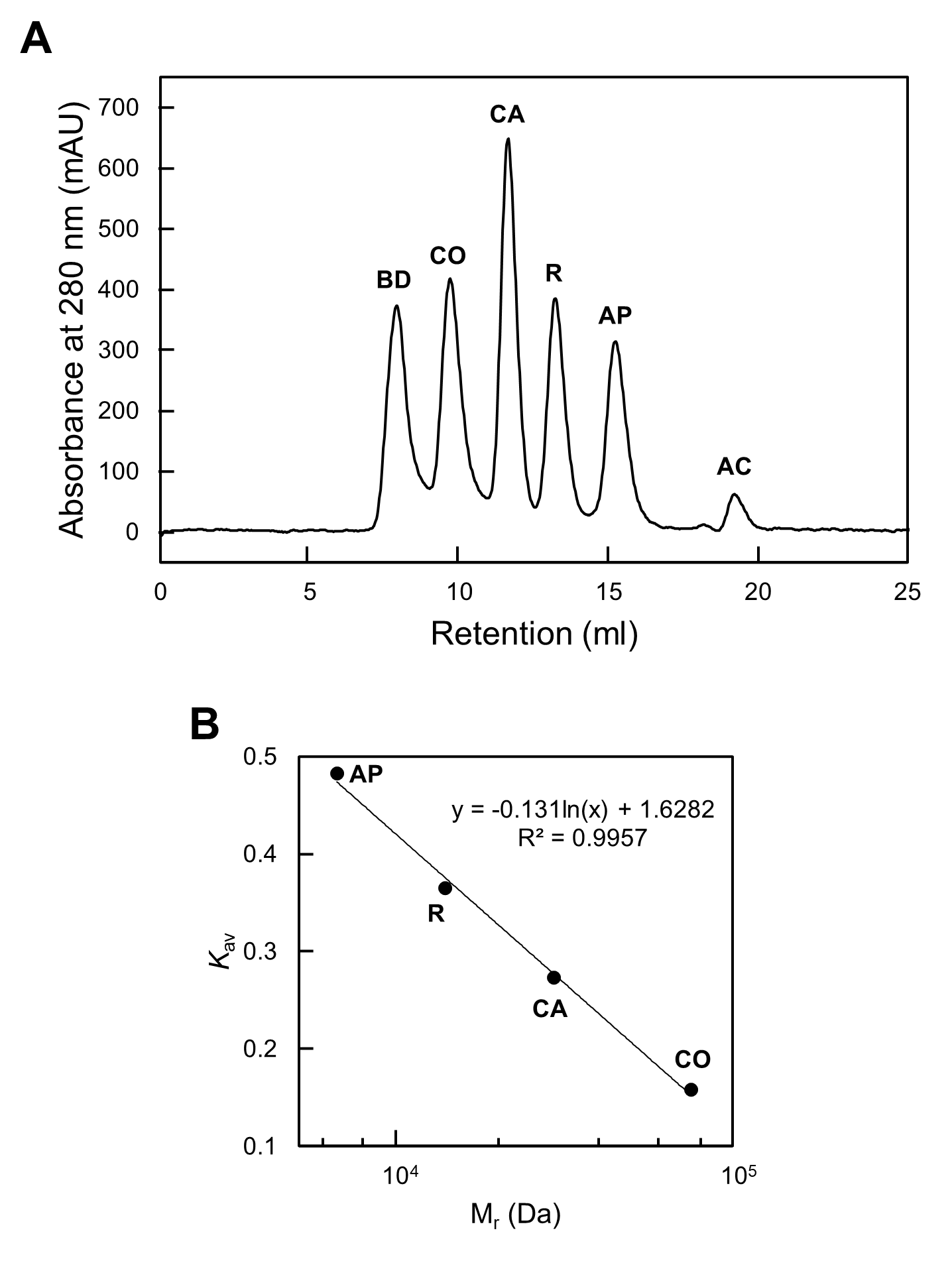

Supplement: Supplementary file 1 [file ijms-25-02157-s001.zip › Quelas_et_al_Figure S4.tif]
